# Supplementary material for: Metabolic reaction network-based recursive metabolite annotation for untargeted metabolomics
Source: Nat Commun. 2019 Apr 3;10:1516. doi: 10.1038/s41467-019-09550-x (PMC6447530; doi:10.1038/s41467-019-09550-x)
Supplement: Supplementary file 1 — Supplementary Information [file 41467_2019_9550_MOESM1_ESM.pdf]

Supplementary information for

Metabolic Reaction Network-based Recursive Metabolite Annotation for Untargeted Metabolomics

Shen *et al.*

List of Supplementary Figures

Supplementary Figure 1: Construction of non-reaction pairs and comparison of dot product, bonanza and hybrid similarity search (HSS) scores.

Supplementary Figure 2: The detailed workflow of recursive metabolite annotation based on Metabolic Reaction Network (MRN).

Supplementary Figure 3: Numbers of neighbor metabolites, annotated metabolites and seed metabolites in each round of recursive annotation (positive mode of *Drosophila* aging dataset).

Supplementary Figure 4: Network diagrams to demonstrate the distributions of annotated metabolites in round 0, round 1, round 2-3, round 4-5, round 6-10 and round 11-19, respectively (positive mode of *Drosophila* aging dataset).

Supplementary Figure 5: Numbers of neighbor metabolites, annotated metabolites and seed metabolites in each round of recursive annotation (negative mode of *Drosophila* aging dataset).

Supplementary Figure 6: Network diagrams to demonstrate the distributions of annotated metabolites in round 0, round 1, round 2-3, round 4-5, round 6-7 and round 8-14, respectively (negative mode of *Drosophila* aging dataset).

Supplementary Figure 7: The optimization of the RT match threshold in MetDNA.

Supplementary Figure 8: The optimization of weight combinations for annotation scores.

Supplementary Figure 9: The coverage and correct annotation rates using different cutoffs for the annotation score.

Supplementary Figure 10: Distribution of the confidence levels for metabolite annotations in *Drosophila* aging dataset: positive mode (a) and negative mode (b).

Supplementary Figure 11: The confirmation of the seed L-Arginine and its neighbor metabolites using chemical standards and online spectral libraries.

Supplementary Figure 12: An example of initial seed metabolite (adenosine diphosphate, ADP) is given to demonstrate how MetDNA annotates 4 neighbor metabolites.

Supplementary Figure 13: Validation of high annotation accuracy from MetDNA using negative mode of mouse liver dataset in validation experiment 1.

Supplementary Figure 14: Validation of high annotation accuracy from MetDNA using negative mode of *Drosophila* aging dataset and *E.coli* dataset in validation experiment 2.

Supplementary Figure 15: Validation of high annotation accuracy from MetDNA using negative mode of *Drosophila* aging dataset in validation experiment 3.

Supplementary Figure 16: The overlap of enriched metabolic pathways using different numbers of seed metabolites.

Supplementary Figure 17: The influence of the initial seed number to the final annotation result (dataset #1-*Drosophila* aging dataset).

Supplementary Figure 18: The influence of the initial seed number to the final annotation result (dataset #2 - aging mouse liver dataset).

Supplementary Figure 19: The influence of the cutoff of annotation score on the numbers of annotated peaks using the correct and four types of misannotated seed metabolites.

Supplementary Figure 20: The dysregulated glycolysis pathway in *Drosophila* aging datasets.

Supplementary Figure 21: The dysregulated arginine biosynthesis pathway in *Drosophila* aging datasets.

List of Supplementary Tables

Supplementary Table 1: The detailed information of 28 metabolites confirmed using chemical standards, METLIN, NIST, HMDB library and CFM-ID.

Supplementary Table 2: The detailed information of all the datasets used to evaluate the performance of MetDNA in this study.

Supplementary Table 3: Metabolite annotation and pathway enrichment analysis results of all the datasets in this study.

Supplementary Table 4: Correct and four types of misannotated seed metabolites in positive mode of *Drosophila* aging dataset (dataset #1).

Supplementary Table 5: Adduct ion table used in MetDNA.

Supplementary Table 6: The detailed parameters for data conversion using ProteoWizard.

Supplementary Table 7: Rules for calculating true positive and true negative rates in validation experiment 1.

Supplementary Table 8: Baseline and histopathologic characteristics of participant subjects in Study 5.

Supplementary Table 9: Baseline and histopathologic characteristics of participant subjects in Study 6.

List of Supplementary Notes

Supplementary Note 1: Experimental details for construction of RPs and non-RPs in our in-house library, NIST and METLIN.

Supplementary Note 2: Experimental details for the MetDNA parameter optimization.

Supplementary Note 3: Experimental details for datasets #2-11.

Supplementary Note 4: Experimental details for validation experiments of MetDNA.

Supplementary Note 5: Experimental details for investigating the influence of tandem spectral library size on the results of MetDNA.

Supplementary Note 6: Experimental details for investigating the propagation of misannotated metabolites.

Supplementary Note 7: Instructions for the processing of *Drosophila* aging dataset using MetDNA.

Supplementary Note 8: Experimental details for annotation of 917 dysregulated peaks in *Drosophila* aging dataset.

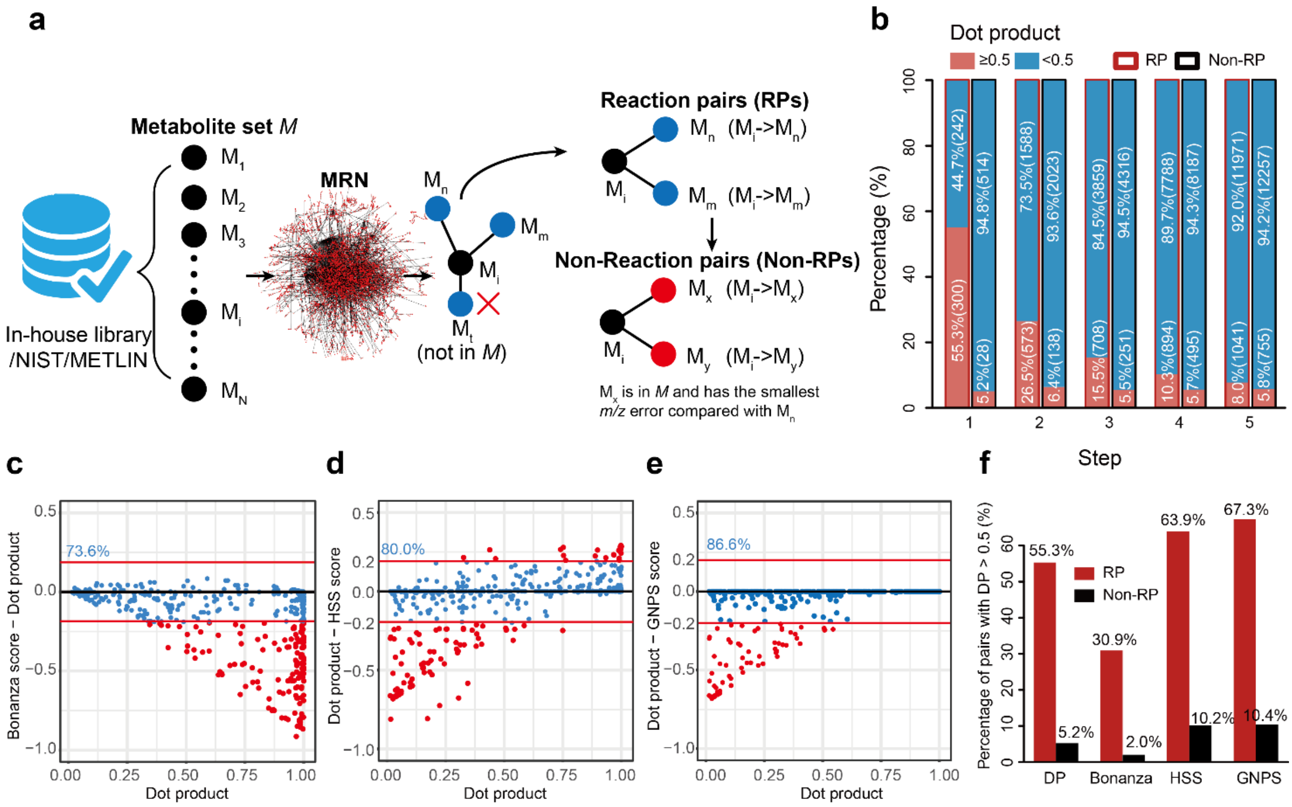

Supplementary Figure 1

Construction of non-reaction pairs and comparison of dot product, bonanza, and hybrid similarity search (HSS) and GNPS scores.

(**a**) Illustration of the construction of reaction pairs (RPs) and non-reaction pairs (non-RPs). (**b**) Spectral similarity between metabolites in RPs and non-RPs with different reaction steps. (**c**) Comparison of dot product (DP) and bonanza scores of metabolites in RPs from in-house library. (**d**) Comparison of dot product and HSS scores of metabolites in RPs from in-house library. (**d**) Comparison of dot product and GNPS scores of metabolites in RPs from in-house library. (**f**) Percentage of pairs with DPs > 0.5 utilizing DP, bonanza, HSS and GNPS scores in the in-house library.

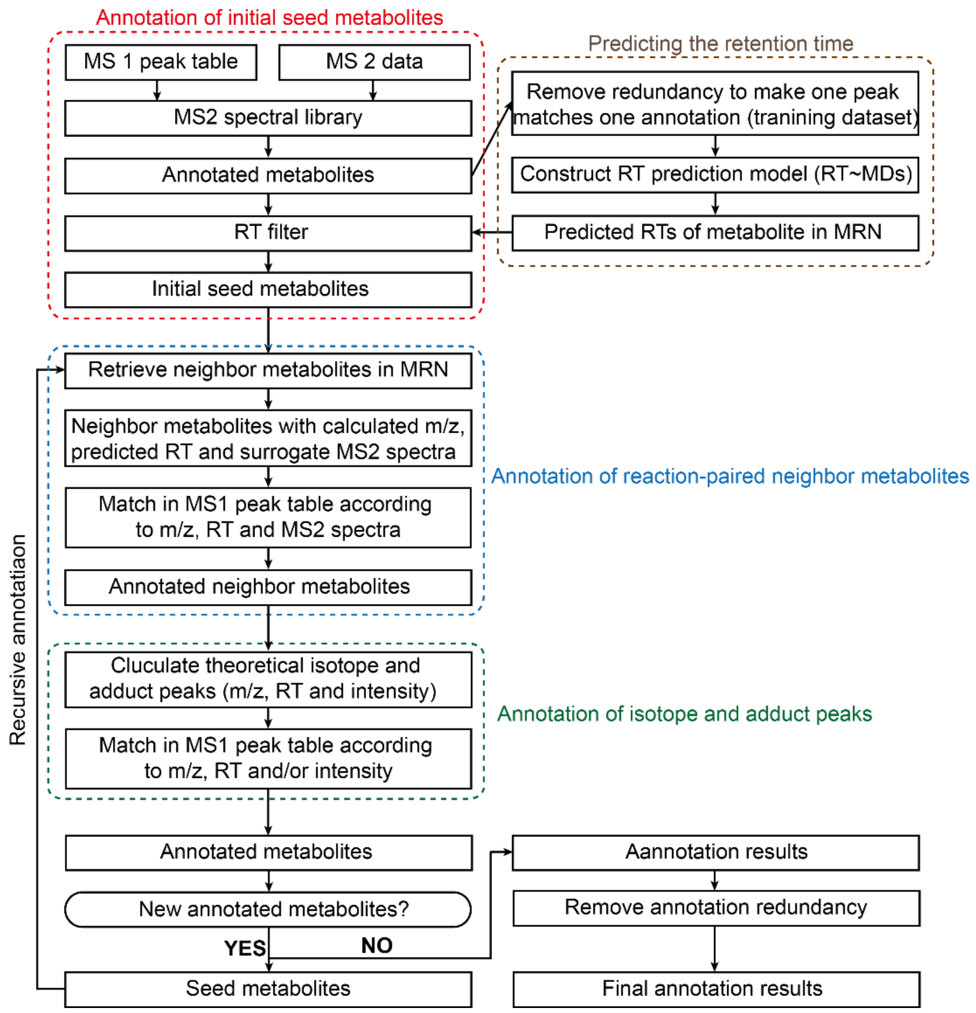

Supplementary Figure 2

The detailed workflow of recursive metabolite annotation based on Metabolic Reaction Network (MRN).

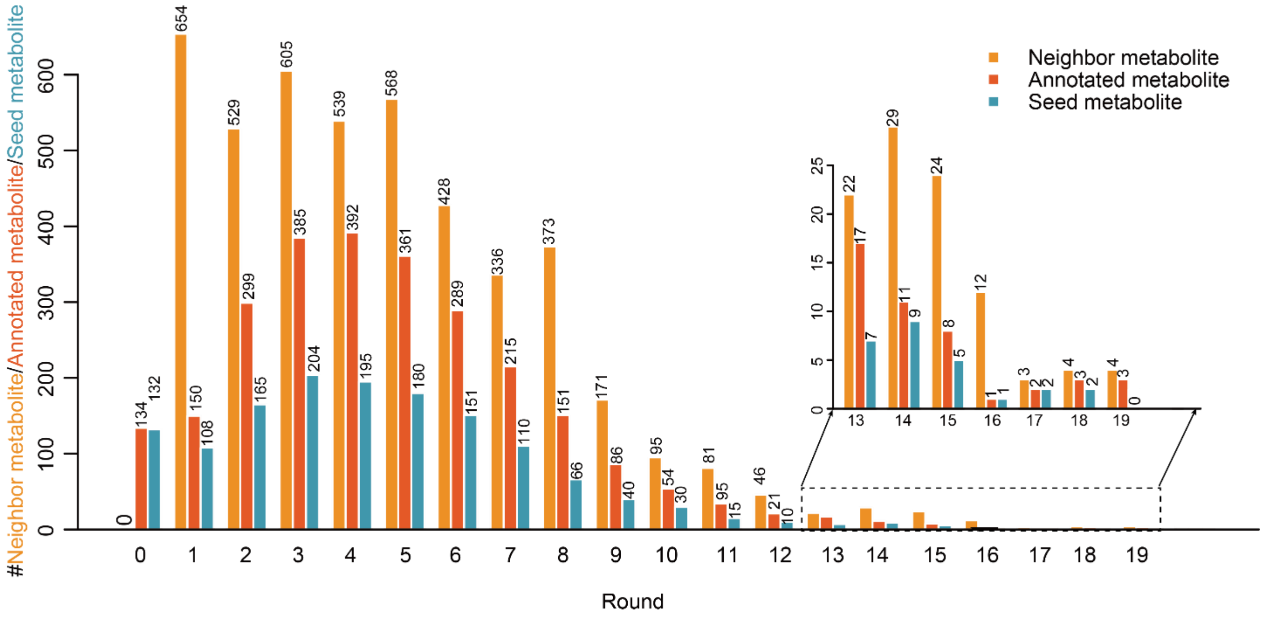

Supplementary Figure 3

Numbers of neighbor metabolites, annotated metabolites and seed metabolites in each round of recursive annotation (positive mode of *Drosophila* aging dataset).

Yellow bar represents the number of neighbor metabolites; red bar represents the number of annotated metabolites; blue bar represents the number of seed metabolites selected from the annotated metabolites. Since metabolites in round 0 are annotated through the MS2 spectral match, there is no neighbor metabolite available. For round 20, since there was no new annotated metabolite, the recursive annotation stopped at round 20.

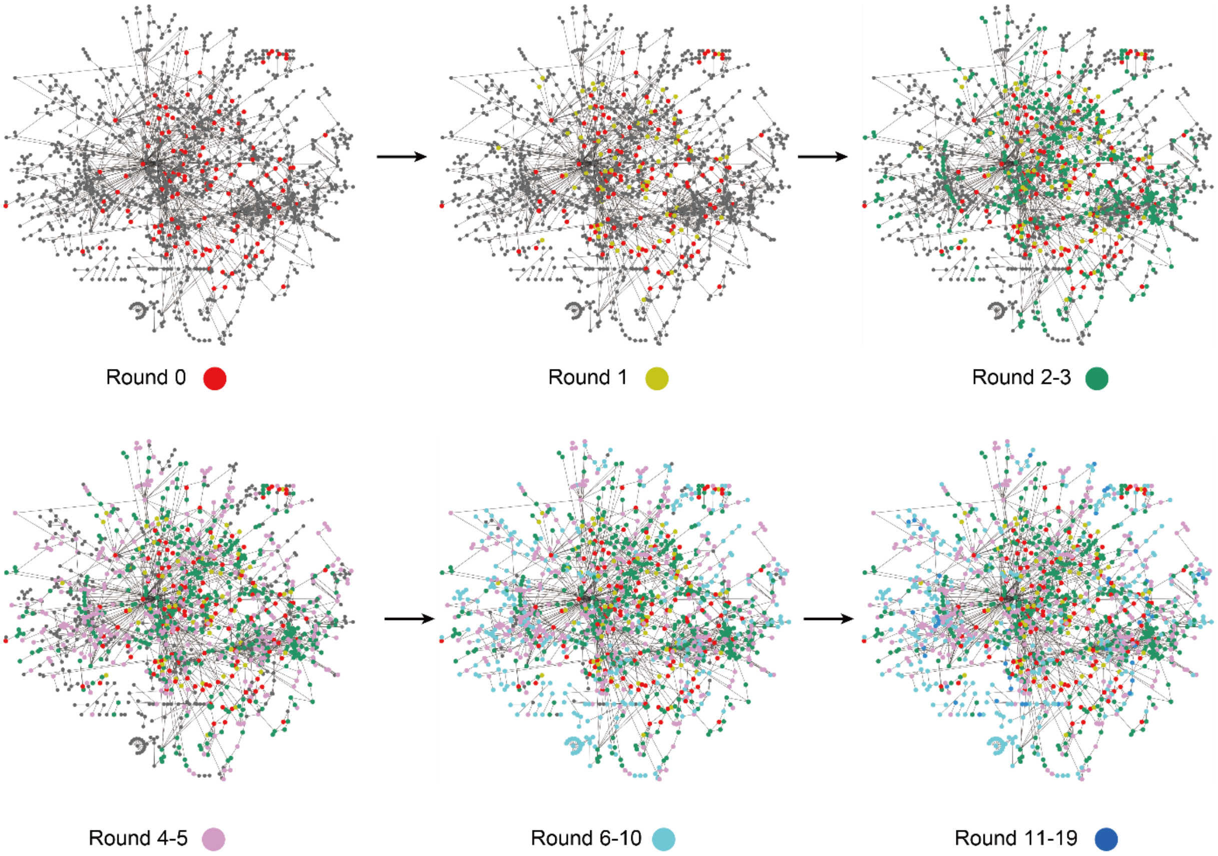

Supplementary Figure 4

Network diagrams to demonstrate the distributions of annotated metabolites in round 0, round 1, round 2-3, round 4-5, round 6-10 and round 11-19, respectively (positive mode of *Drosophila* aging dataset).

A total of 1,496 metabolites were annotated through the 19 rounds of recursive annotation. In each network diagram, annotated metabolites are labeled as color dots, while unannotated ones are labeled as gray dots.

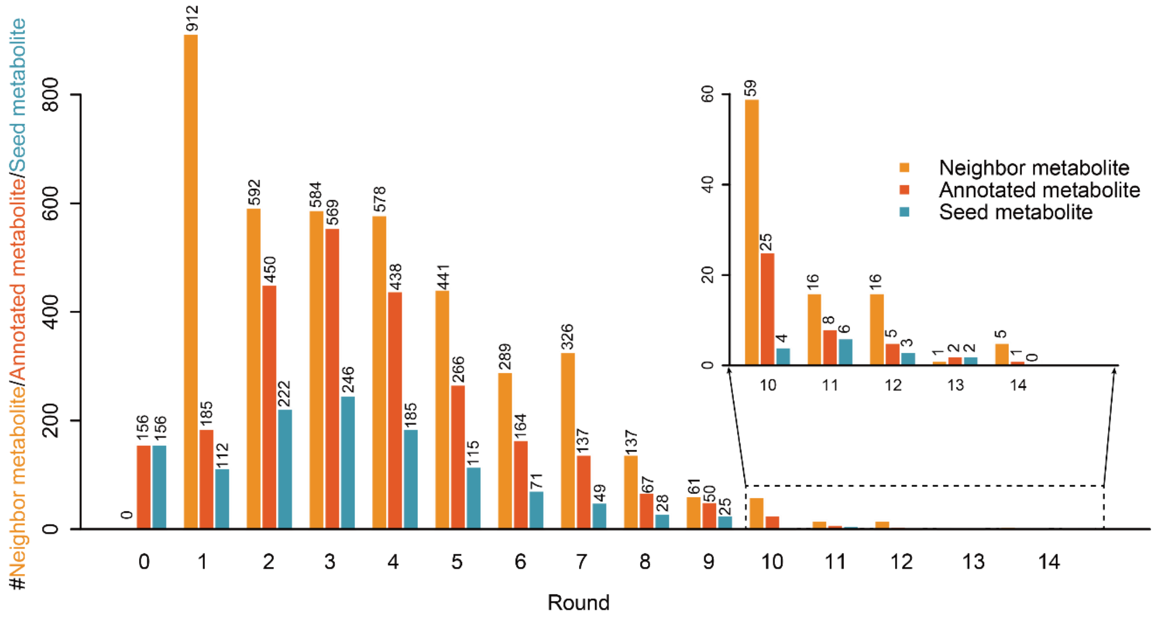

Supplementary Figure 5

Numbers of neighbor metabolites, annotated metabolites and seed metabolites in each round of recursive annotation (negative mode of *Drosophila* aging dataset).

Yellow bar represents the number of neighbor metabolites; red bar represents the number of annotated metabolites; blue bar represents the number of seed metabolites selected from the annotated metabolites. Since metabolites in round 0 are annotated through the MS2 spectral match, there is no neighbor metabolite available. For round 14, since there was no new annotated metabolite, the recursive annotation stopped at round 14.

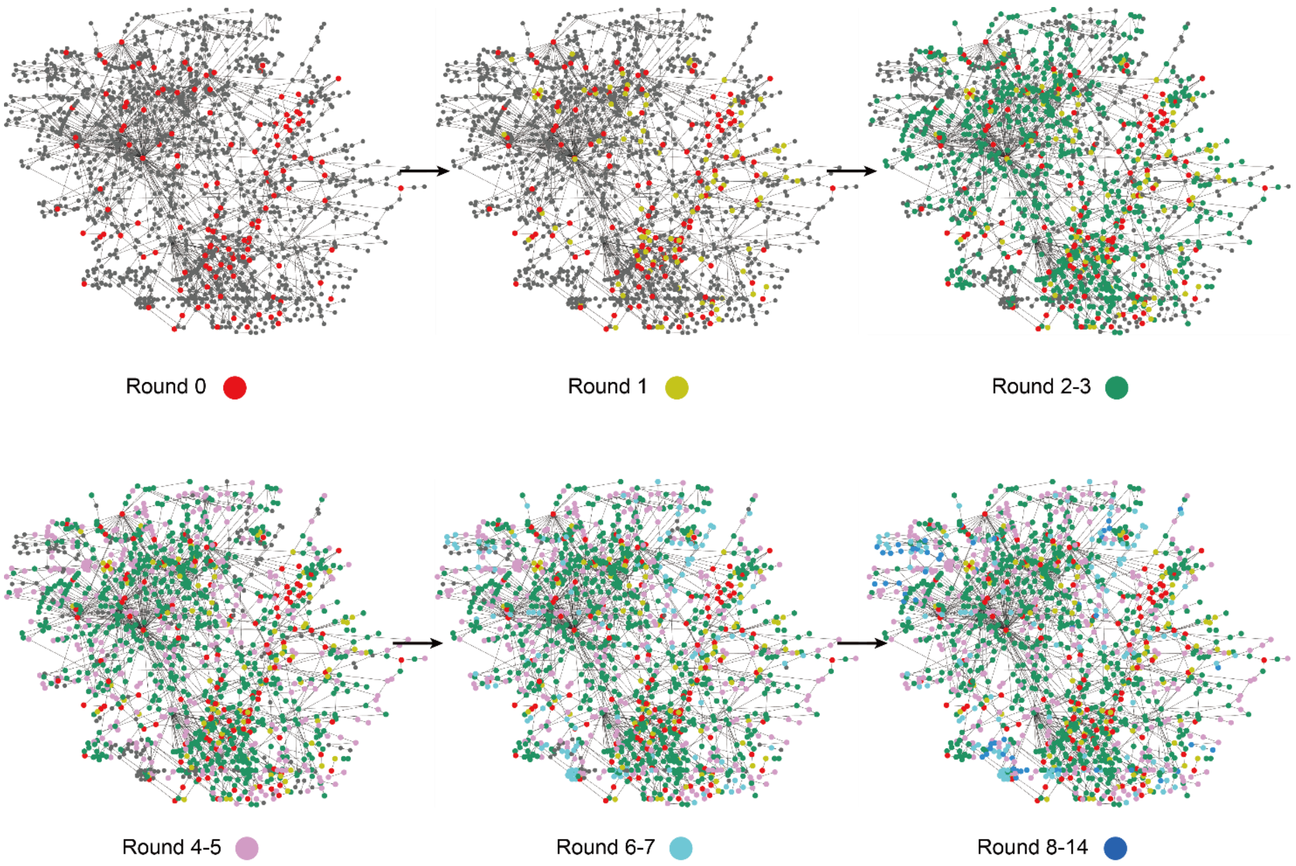

Supplementary Figure 6

Network diagrams to demonstrate the distributions of annotated metabolites in round 0, round 1, round 2-3, round 4-5, round 6-7 and round 8-14, respectively (negative mode of *Drosophila* aging dataset).

A total of 1,538 metabolites were annotated through the 14 rounds of recursive annotation. In each network diagram, annotated metabolites are labeled as color dots, while unannotated ones are labeled as gray dots.

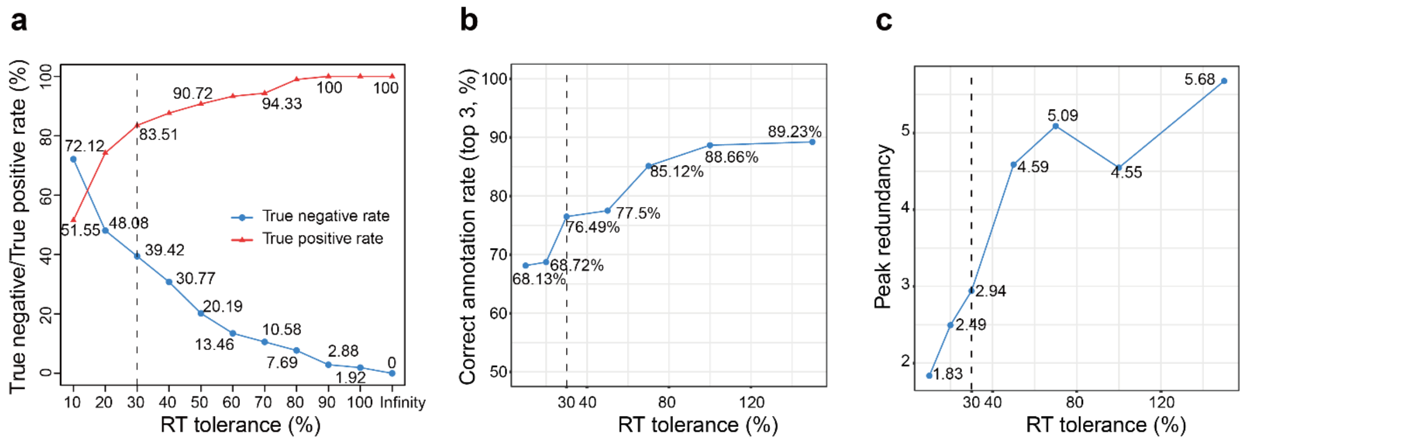

Supplementary Figure 7

The optimization of the RT match threshold in MetDNA.

(**a**) The true positive and true negative rates using different RT match thresholds in experiment 1 in Supplementary Note 2. (**b**) The correct annotation rates using different RT match thresholds in experiment 2 in Supplementary Note 2. (**c**) The peak redundancies using different RT match thresholds in experiment 2.

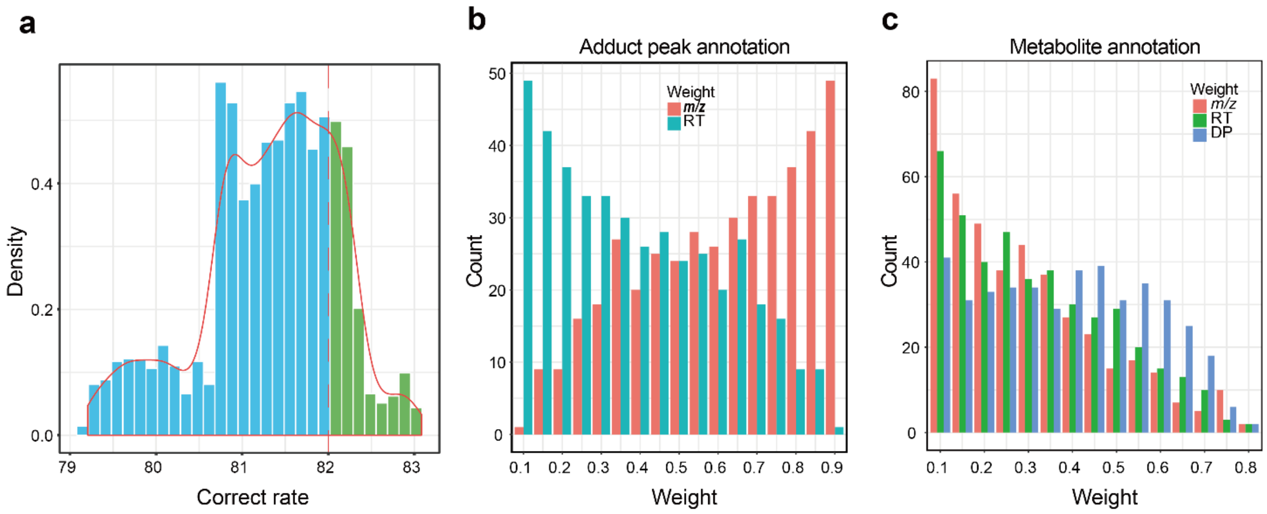

Supplementary Figure 8

The optimization of weight combinations for annotation scores.

(**a**) The distribution of correct annotation rates with different weight combinations. (**b**) The distribution of weight combination for adduct peak annotation (i.e., for m/z match and RT match scores) with high correct annotation rates. (**c**) The distribution of weight combination for metabolite annotation (i.e., for m/z match, RT match and MS2 spectral match scores) with high correct annotation rates.

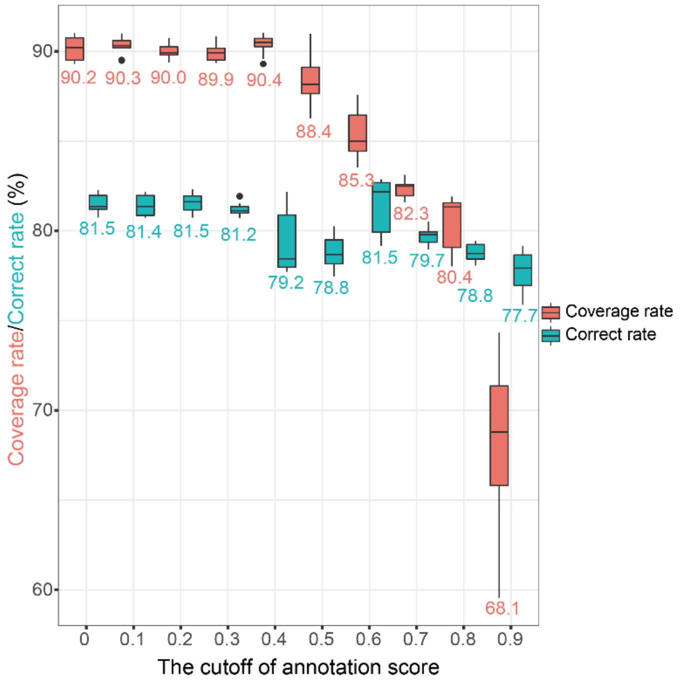

Supplementary Figure 9

The coverage and correct annotation rates using different cutoffs for the annotation score.

The x-axis represents the cutoff of the annotation score. The y-axis represents the coverage rate (red) and correct rate (green). The upper, middle and lower lines correspond to the first, second and third quartiles (the 25th, 50th and 75th percentiles).

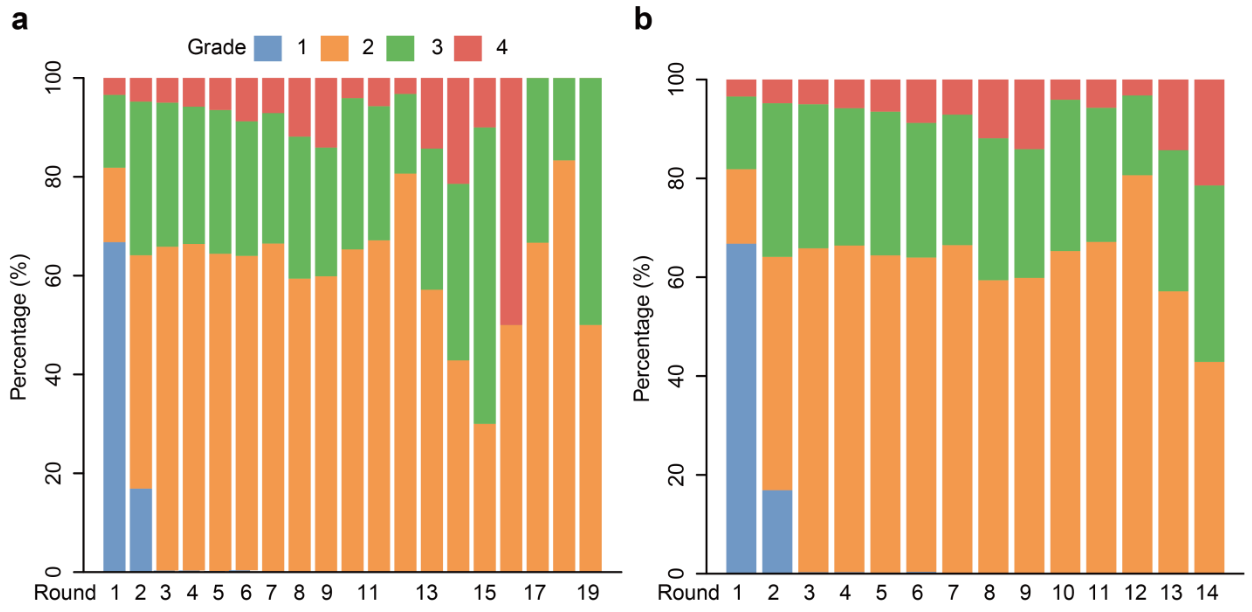

Supplementary Figure 10

Distribution of the confidence levels for metabolite annotations in *Drosophila* aging dataset: positive mode (a) and negative mode (b).

Blue, yellow, green and red bars represent the confidence levels of metabolite annotations from grades 1, 2, 3 to 4, respectively.

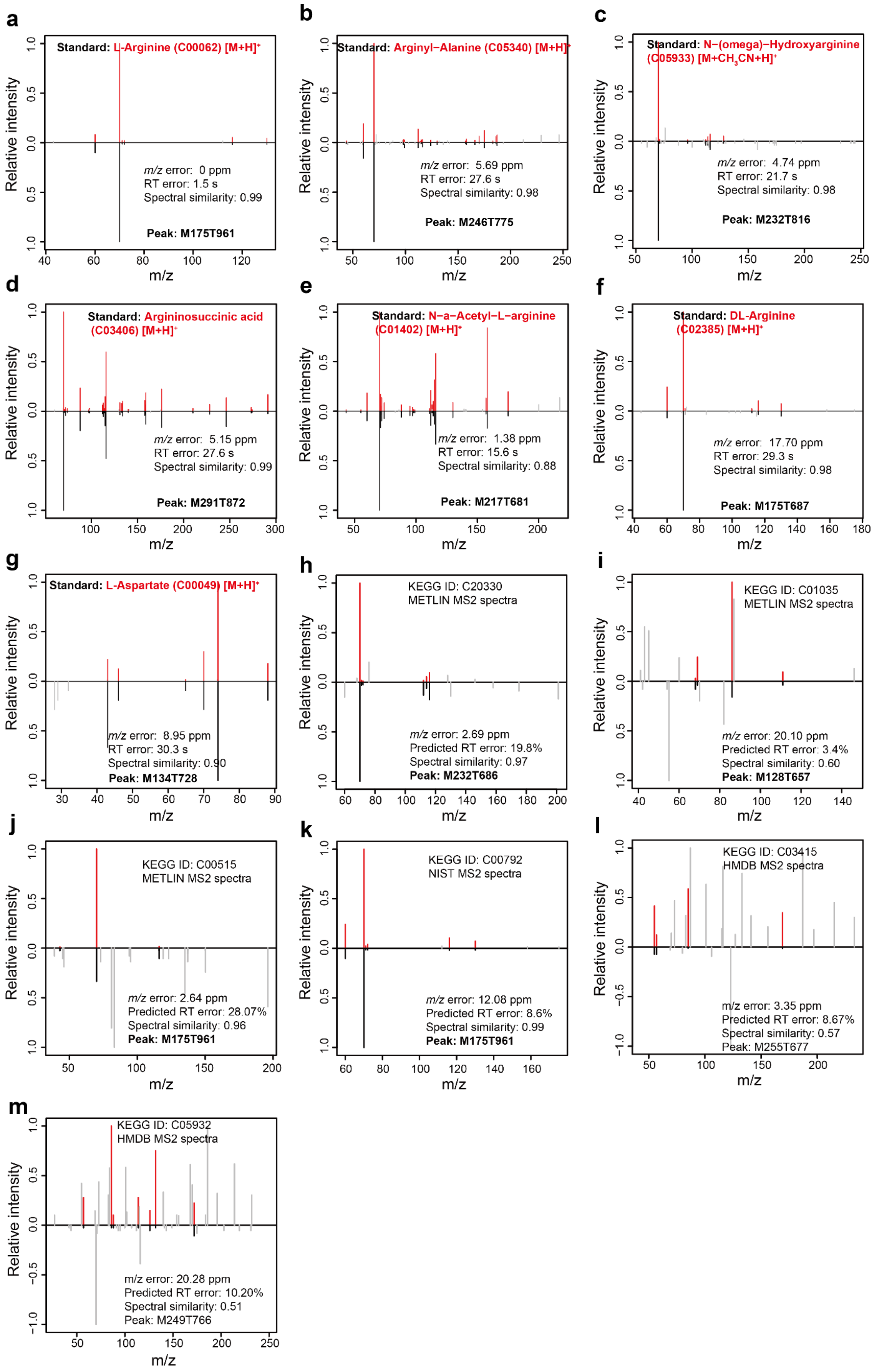
a
Standard: L-Arginine (C00062) [M+H]+
m/z error: 0 ppm
RT error: 1.5 s
Spectral similarity: 0.99
Peak: M175T961
b
Standard: Arginyl−Alanine (C05340) [M+H]+
m/z error: 5.69 ppm
RT error: 27.6 s
Spectral similarity: 0.98
Peak: M246T775
c
Standard: N−(omega)−Hydroxyarginine (C05933) [M+CH3CN+H]+
m/z error: 4.74 ppm
RT error: 21.7 s
Spectral similarity: 0.98
Peak: M232T816
d
Standard: Argininosuccinic acid (C03406) [M+H]+
m/z error: 5.15 ppm
RT error: 27.6 s
Spectral similarity: 0.99
Peak: M291T872
e
Standard: N−a−Acetyl−L−arginine (C01402) [M+H]+
m/z error: 1.38 ppm
RT error: 15.6 s
Spectral similarity: 0.88
Peak: M217T681
f
Standard: DL-Arginine (C02385) [M+H]+
m/z error: 17.70 ppm
RT error: 29.3 s
Spectral similarity: 0.98
Peak: M175T687
g
Standard: L-Aspartate (C00049) [M+H]+
m/z error: 8.95 ppm
RT error: 30.3 s
Spectral similarity: 0.90
Peak: M134T728
h
KEGG ID: C20330
METLIN MS2 spectra
m/z error: 2.69 ppm
Predicted RT error: 19.8%
Spectral similarity: 0.97
Peak: M232T686
i
KEGG ID: C01035
METLIN MS2 spectra
m/z error: 20.10 ppm
Predicted RT error: 3.4%
Spectral similarity: 0.60
Peak: M128T657
j
KEGG ID: C00515
METLIN MS2 spectra
m/z error: 2.64 ppm
Predicted RT error: 28.07%
Spectral similarity: 0.96
Peak: M175T961
k
KEGG ID: C00792
NIST MS2 spectra
m/z error: 12.08 ppm
Predicted RT error: 8.6%
Spectral similarity: 0.99
Peak: M175T961
l
KEGG ID: C03415
HMDB MS2 spectra
m/z error: 3.35 ppm
Predicted RT error: 8.67%
Spectral similarity: 0.57
Peak: M255T677
m
KEGG ID: C05932
HMDB MS2 spectra
m/z error: 20.28 ppm
Predicted RT error: 10.20%
Spectral similarity: 0.51
Peak: M249T766
Relative intensity
m/z

Supplementary Figure 11

The confirmation of the seed L-Arginine and its neighbor metabolites using chemical standards and online spectral libraries.

(**a**) The annotation of peak M175T961 (L-Arginine) is confirmed using the chemical standard with m/z error of 0 ppm, RT error of 1.5 s and MS2 spectral similarity of 0.99. (**b**) The annotation of peak M246T775 (Arginyl-Alanine) is confirmed using the chemical standard with m/z error of 5.69 ppm, RT error of 27.6 s and MS2 spectral similarity of 0.98. (**c**) The annotation of peak M232T816 (N-(omega)-Hydroxyarginine) is confirmed using chemical standard with m/z error of 4.74 ppm, RT error of 21.7 s and MS2 spectral similarity of 0.98. (**d**) The annotation of peak M291T872 (Argininosuccinic acid) is confirmed using the chemical standard with m/z error of 5.15 ppm, RT error of 27.6 s and MS2 spectral similarity of 0.99. (**e**) The annotation of peak M217T681 (N-alpha-Acetyl-L-arginine) is confirmed using the chemical standard with m/z error of 1.38 ppm, RT error of 15.6 s and MS2 spectral similarity of 0.88. (**f**) The annotation of peak M175T687 (DL-Arginine) is confirmed using chemical standard with m/z error of 17.70 ppm, RT error of 29.3 s and MS2 spectral similarity of 0.98. (**g**) The annotation of peak M134T728 (L-Aspartate) is confirmed using the chemical standard with m/z error of 8.95 ppm, RT error of 30.3 s and MS2 spectral similarity of 0.90. Red represents the standard MS2 spectrum from the standard and black represents the experimental MS2 spectrum. (**h-m**) Six metabolites were validated using METLIN, NIST or HMDB libraries. The RT cutoff was set as 30%.

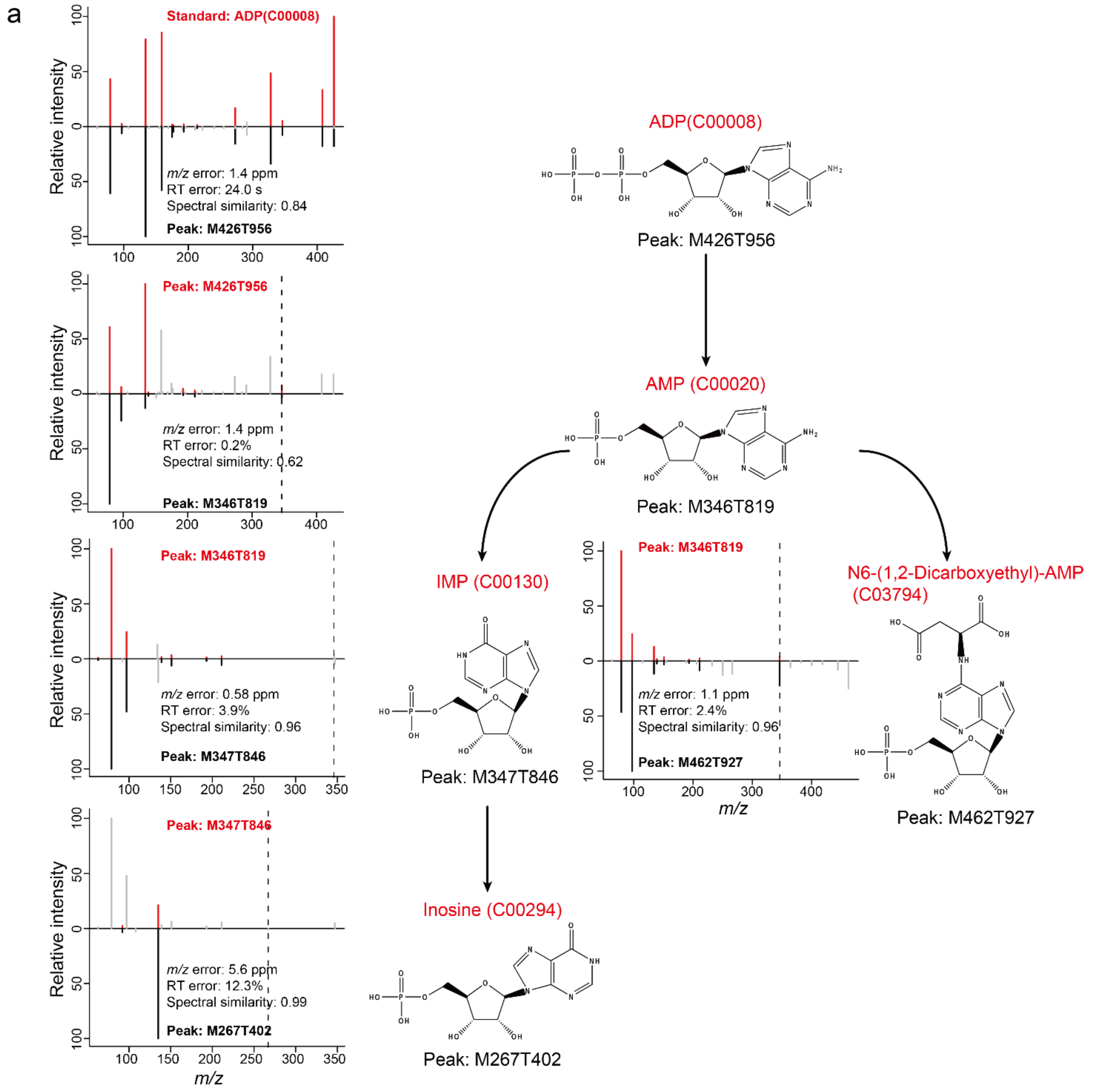

Supplementary Figure 12

An example of initial seed metabolite (adenosine diphosphate, ADP) is given to demonstrate how MetDNA annotates 4 neighbor metabolites.

(**a**) The use of initial seed metabolite (ADP) to annotate 4 neighbor metabolites: adenosine diphosphate (AMP); inosine monophosphate (IMP); N6-(1, 2-Dicarboxyethyl)-AMP and inosine. Peak M426T956 (m/z 426.021, retention time 956.2 s) was annotated as ADP (C00008) using the in-house library with a m/z error of 1.4 ppm, retention time (RT) error of 24.0 s and a MS2 spectral similarity score (dot-product score) of 0.84 (top left). Then, ADP was used as a seed metabolite and its MS2 spectrum was assigned to one of its neighbor metabolites (AMP, C00020) as surrogate MS2 spectrum. Peak M346T819 (m/z 346.0558, retention time 819.3 s) was annotated as AMP with a DP score of 0.62, m/z error of 1.4 ppm, RT error of 0.2%. Then, AMP was

selected as a new seed metabolite and its MS2 spectrum was assigned to two of its neighbor metabolites (IMP, C00130; N6-(1, 2-Dicarboxyethyl)-AMP, C03794) as the surrogate MS2 spectrum. Peak M347T846 (m/z 347.0395, retention time 845.8 s) was annotated as IMP with a DP score of 0.96, m/z error of 0.58 ppm, RT error of 3.9%. Peak M462T927 (m/z 462.0657, retention time 927.1 s) was annotated as N6-(1, 2-Dicarboxyethyl)-AMP with a DP score of 0.62, m/z error of 1.1 ppm, RT error of 2.4%. Then IMP was selected as a new seed metabolite and its MS2 spectrum was assigned to one of its neighbor metabolites (inosine, C00294) as surrogate MS2 spectrum. Peak M267T402 (m/z 267.0744, retention time 402.4 s) was annotated as inosine with a DP score of 0.99, m/z error of 5.6 ppm, RT error of 12.3%.

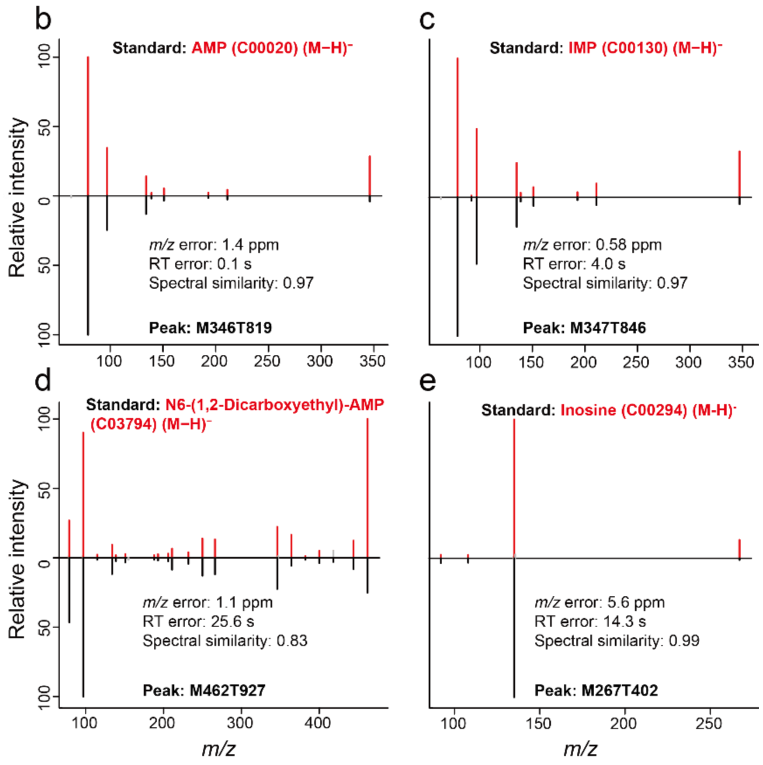

Supplementary Figure 12 continued

(**b**) The annotation of peak M346T819 (AMP) is confirmed using the chemical standard with m/z error of 1.4 ppm, RT error of 0.1 s and MS2 spectral similarity of 0.97. (**c**) The annotation of peak M347T846 (IMP) is confirmed using the chemical standard with m/z error of 0.58 ppm, RT error of 4.0 s and MS2 spectral similarity of 0.97. (**d**) The annotation of peak M462T927 (N6-(1, 2-Dicarboxyethyl)-AMP) is confirmed using chemical standard metabolite with m/z error of 1.1 ppm, RT error of 25.6 s and MS2 spectral similarity of 0.83. (**e**) The annotation of peak M267T402 (inosine) is confirmed using the chemical standard with m/z error of 5.6 ppm, RT error of 14.3 s and MS2 spectral similarity of 0.99. Red represents the standard MS2 spectrum form the standard and black represents the experimental MS2 spectrum.

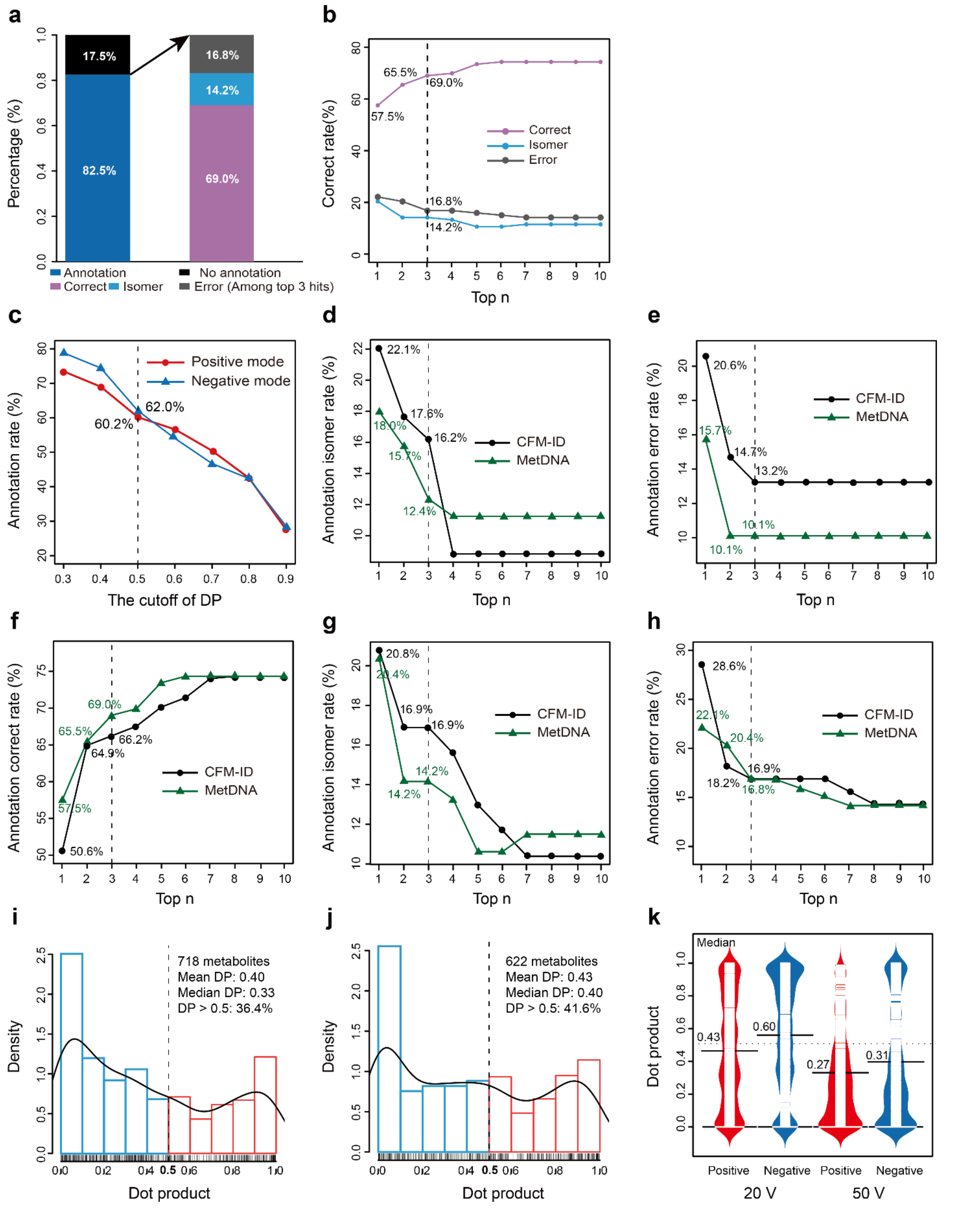

Supplementary Figure 13

Validation of high annotation accuracy from MetDNA using positive and negative mode of mouse liver dataset in validation experiment 1.

(**a**) Statistics of the annotation coverage, and correct, isomer and error rates among the top 3 candidates; (**b**)

statistics of correct, isomer and error rates among top n (n=1 to 10) annotations. The x-axis represents the top n (n=1-10) metabolite candidates for each peak. The y-axis represents the percentages for correct (purple), isomeric (blue) and erroneous (black) annotations.

(**c**)The annotation rates for *in silico* MS2 spectral match with different DP cutoffs. (**d-e**) Comparison of annotation isomeric rate (**d**) and erroneous rate (**e**) between CFM-ID and MetDNA in the positive mode dataset (validation experiment 1).

(**f-h**) Comparison of annotation correct (**f**), isomeric (**g**) and erroneous rate (**h**) between CFM-ID and MetDNA in the negative mode dataset (validation experiment 1).

(**i-j**) The distributions of DP scores for the spectral similarities between the standard MS2 spectra and *in silico* MS2 spectra in positive (**i**) and negative modes (**j**). The CE is 30 eV.

(**k**) The distributions of DP scores for the spectral similarities between the standard MS2 spectra and *in silico* MS2 spectra with positive and negative mode. The CE are 20 eV and 50 eV.

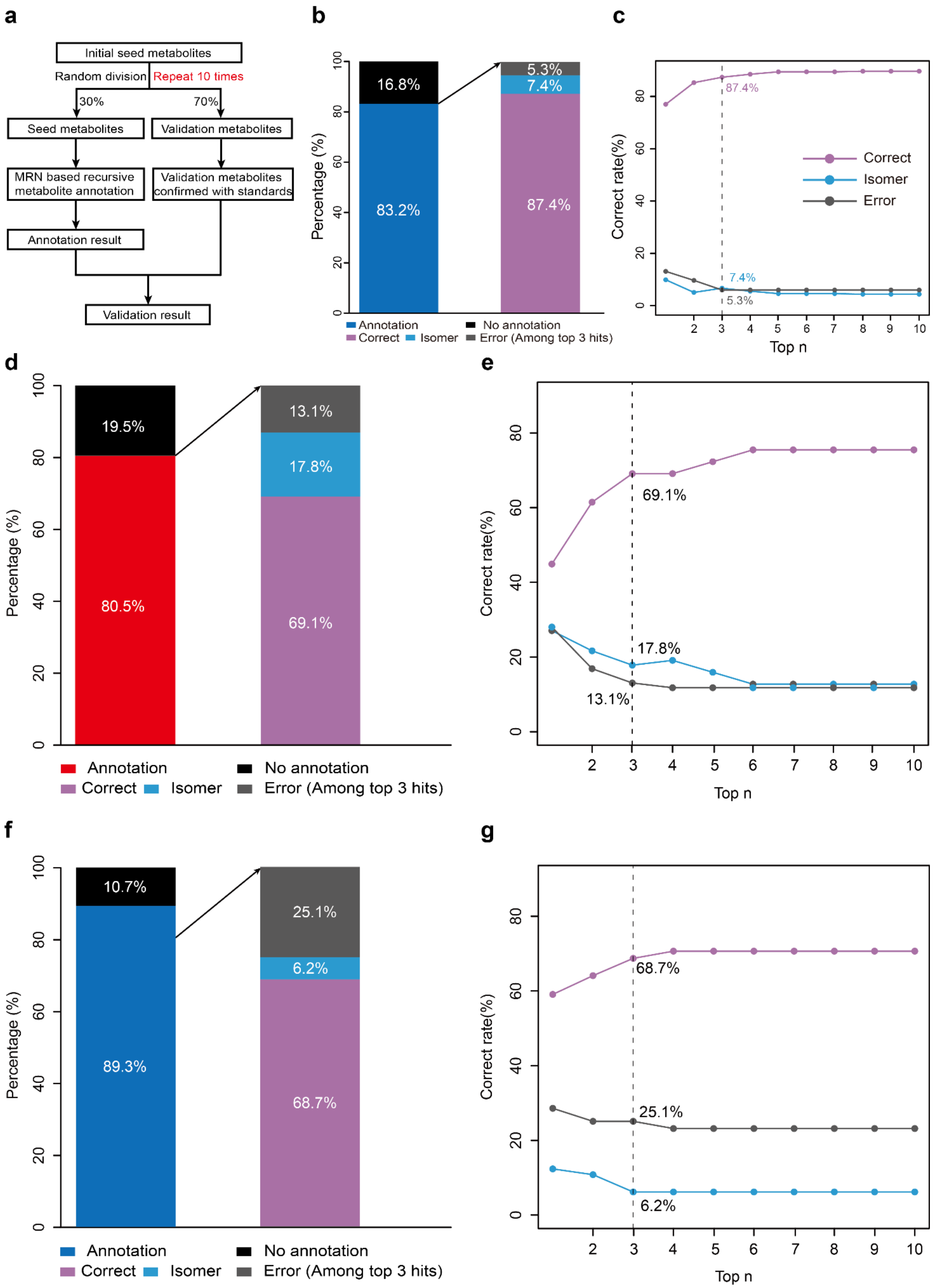
a
Initial seed metabolites
Random division
Repeat 10 times
30%
70%
Seed metabolites
Validation metabolites
MRN based recursive metabolite annotation
Validation metabolites confirmed with standards
Annotation result
Validation result
b
16.8%
83.2%
5.3%
7.4%
87.4%
Percentage (%)
Annotation
No annotation
Correct
Isomer
Error (Among top 3 hits)
c
Correct rate(%)
87.4%
7.4%
5.3%
Correct
Isomer
Error
Top n
d
19.5%
80.5%
13.1%
17.8%
69.1%
Percentage (%)
Annotation
No annotation
Correct
Isomer
Error (Among top 3 hits)
e
69.1%
17.8%
13.1%
Correct rate(%)
Top n
f
10.7%
89.3%
25.1%
6.2%
68.7%
Percentage (%)
Annotation
No annotation
Correct
Isomer
Error (Among top 3 hits)
g
68.7%
25.1%
6.2%
Correct rate(%)
Top n

Supplementary Figure 14

Validation of high annotation accuracy from MetDNA using negative mode of *Drosophila* aging dataset and *E.coli* dataset in validation experiment 2.

(**a**) Design for validation experiment 2. (**b, c**) Validation of metabolite annotations in negative mode of *Drosophila* aging dataset. (**d-g**) Validation of metabolite annotations in positive (**d, e**) and negative (**f, g**) modes of *E.coli* datasets. (**b, d, f**) Statistics of the annotation coverage, and correct, isomer and error rates among the top 3 candidates; (**c, e, g**) statistics of correct, isomer and error rates among top n (n=1 to 10) annotations. The x-axis represents the top n (n=1-10) metabolite candidates for each peak. The y-axis represents the percentages for correct (purple), isomeric (blue) and erroneous (black) annotations.

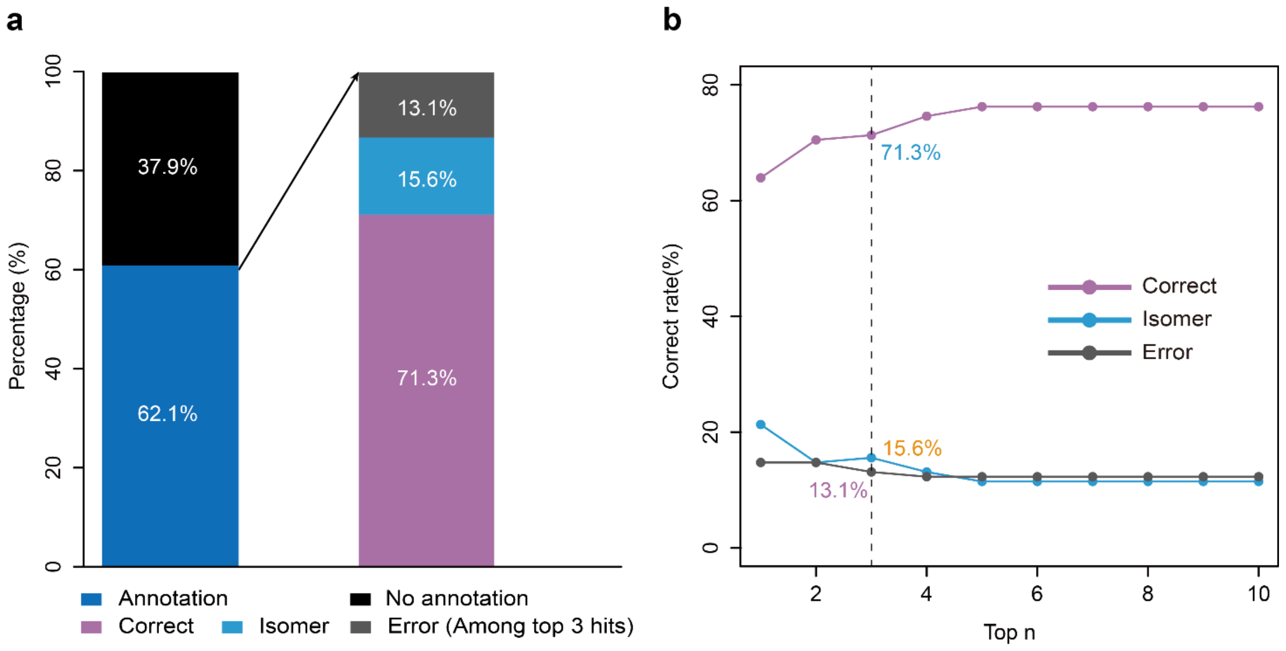

Supplementary Figure 15

Validation of high annotation accuracy from MetDNA using negative mode of *Drosophila* aging dataset in validation experiment 3.

(**a**) Statistics of the annotation coverage, and correct, isomer and error rates among the top 3 candidates; (**b**) statistics of correct, isomer and error rates among top n (n=1 to 10) annotations. The x-axis represents the top n (n=1-10) metabolite candidates for each peak. The y-axis represents the percentages for correct (purple), isomeric (blue) and erroneous (black) annotations.

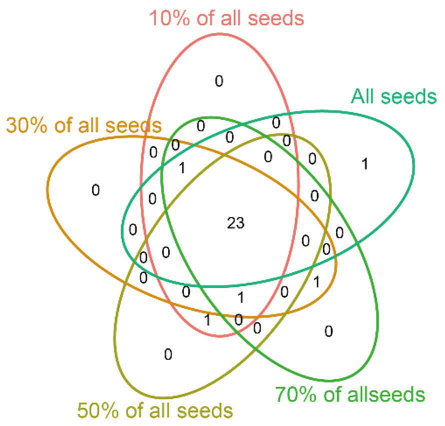

Supplementary Figure 16

The overlap of enriched metabolic pathways using different numbers of seed metabolites.

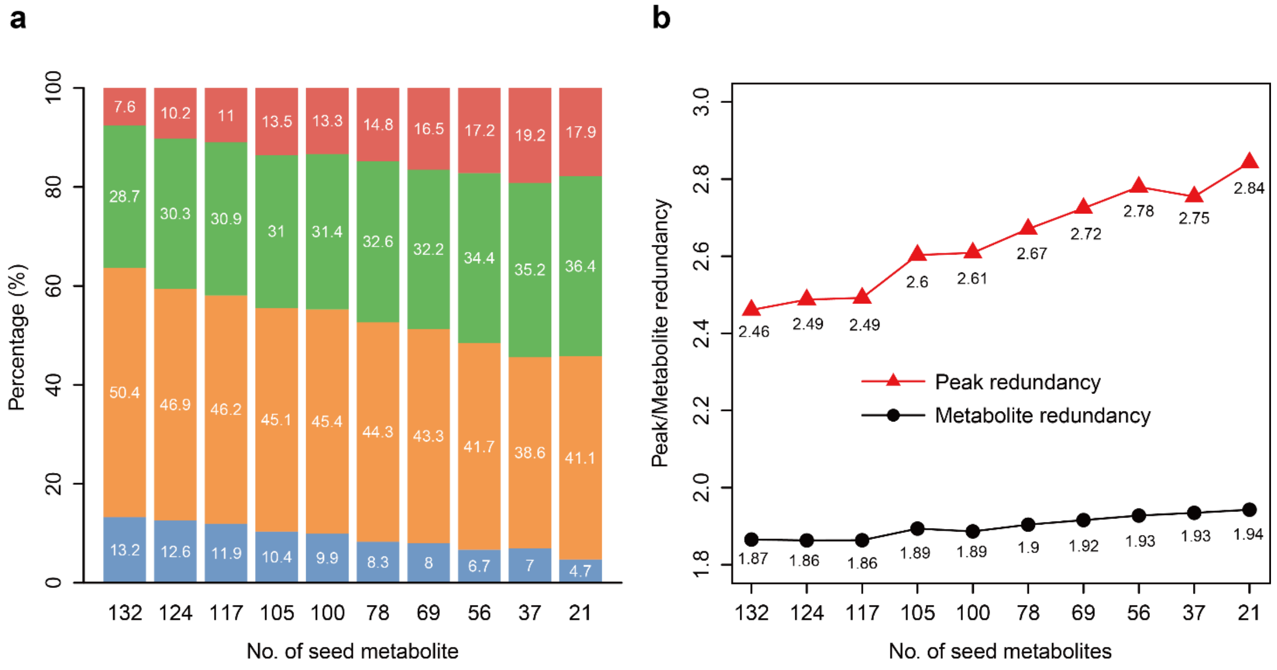

Supplementary Figure 17

The influence of the initial seed number to the final annotation result (dataset #1- *Drosophila* aging dataset).

(**a**) The distributions of the confidence levels for metabolite annotations using different numbers of initial seed metabolites (132, 124, 117, 105, 100, 78, 69, 56, 37, and 21). (**b**) The statistics for peak redundancy and metabolite redundancy using different numbers of initial seed metabolites. All top 5 ranked metabolite annotations with score larger than 0.4 were reserved for the statistics.

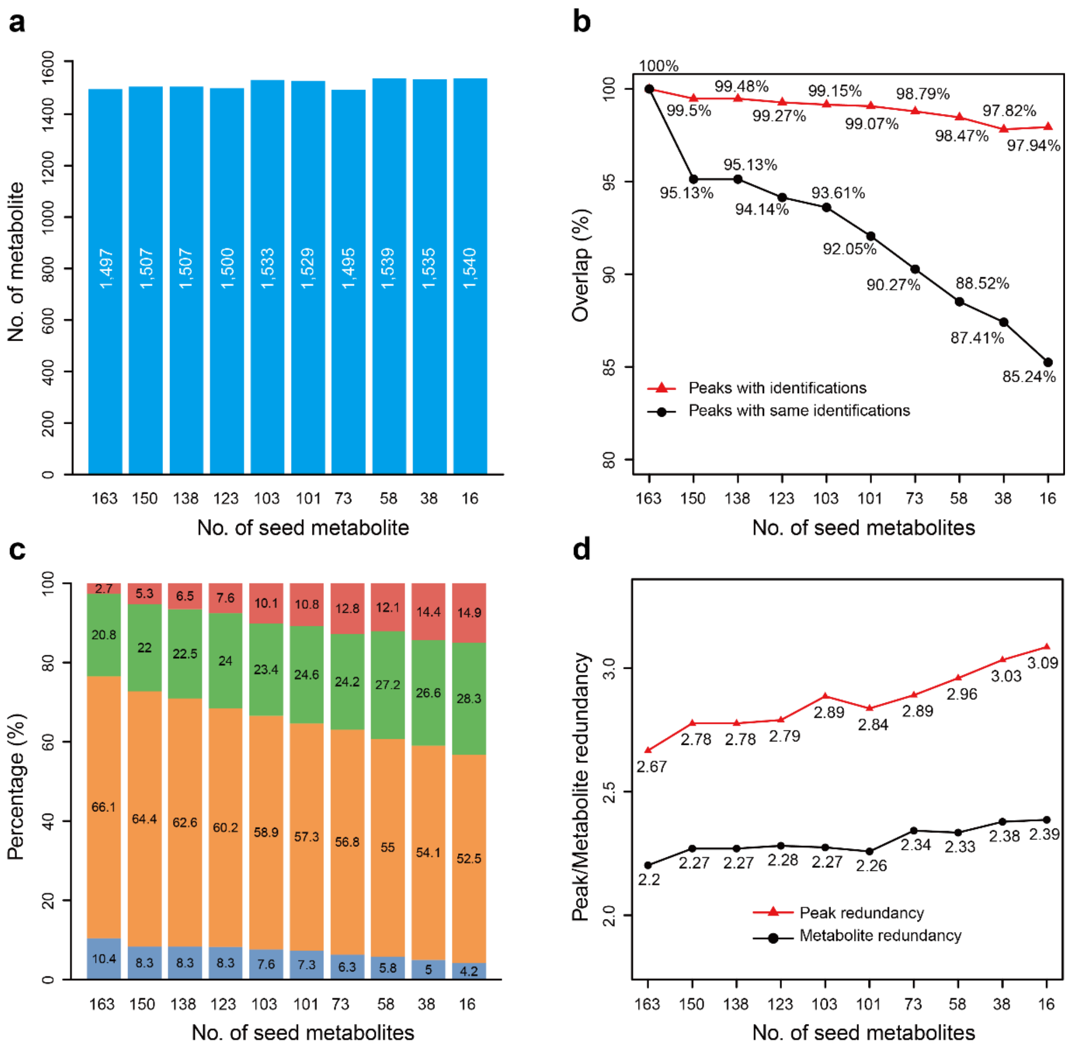

Supplementary Figure 18

The influence of the initial seed number to the final annotation result (dataset #2 - aging mouse liver dataset).

(a) The numbers of annotated metabolites using different numbers of seed metabolites. **(b)** Consistency of annotation results using different numbers of seed metabolites. **(c)** The distributions of the confidence levels for metabolite annotations using different numbers of seed metabolites. **(d)** The statistics for peak redundancy and metabolite redundancy using different numbers of seed metabolites.

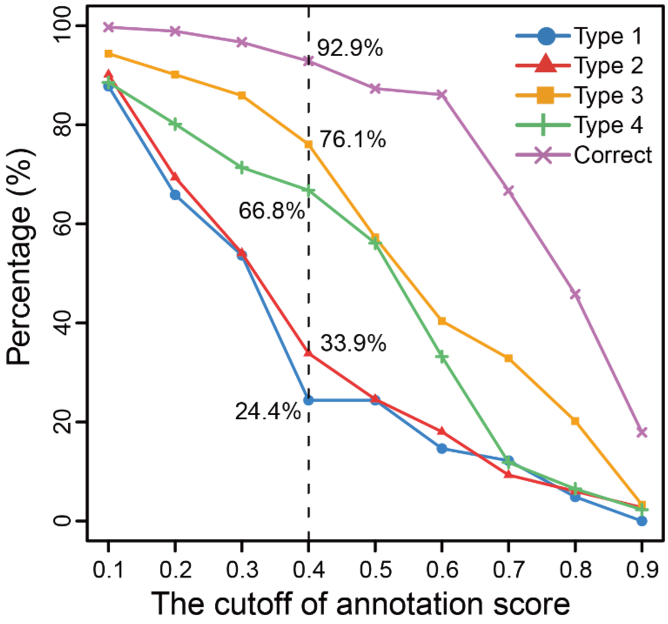

Supplementary Figure 19

The influence of the cutoff of annotation score on the numbers of annotated peaks using the correct and four types of misannotated seed metabolites.

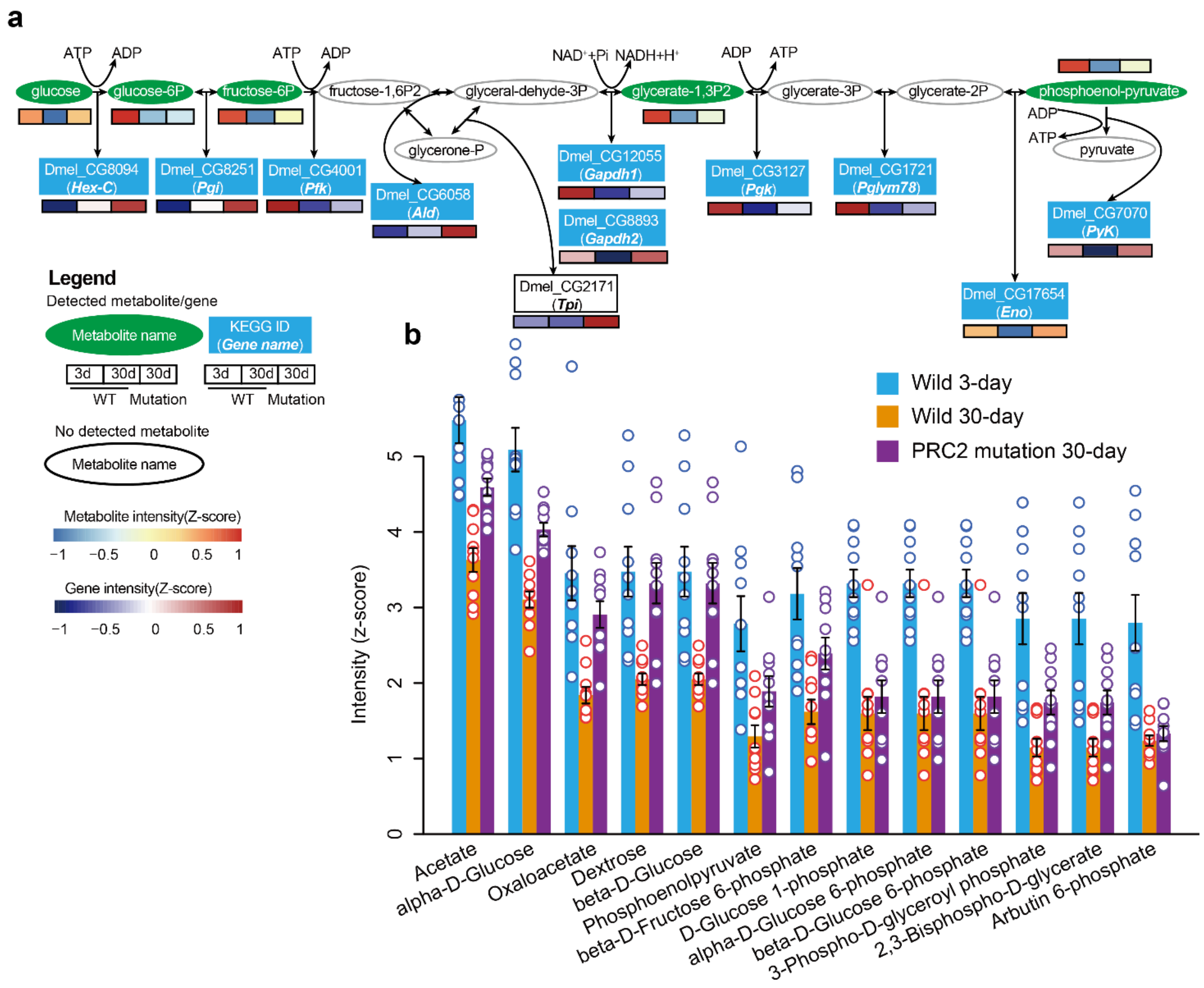

Supplementary Figure 20

The dysregulated glycolysis pathway in *Drosophila* aging datasets.

(**a**) Diagram illustrates that the transcriptional and metabolic changes in glycolysis in 3d- and 30d-old WT and PRC2 long-lived mutants fruit flies. (**b**) Barplots show thirteen detected metabolites in 3d- and 30d-old WT and long-lived PRC2 mutant fruit flies. The y-axis represents the z-score of metabolites (mean ± SD), n = 10 biologically independent samples for each group.

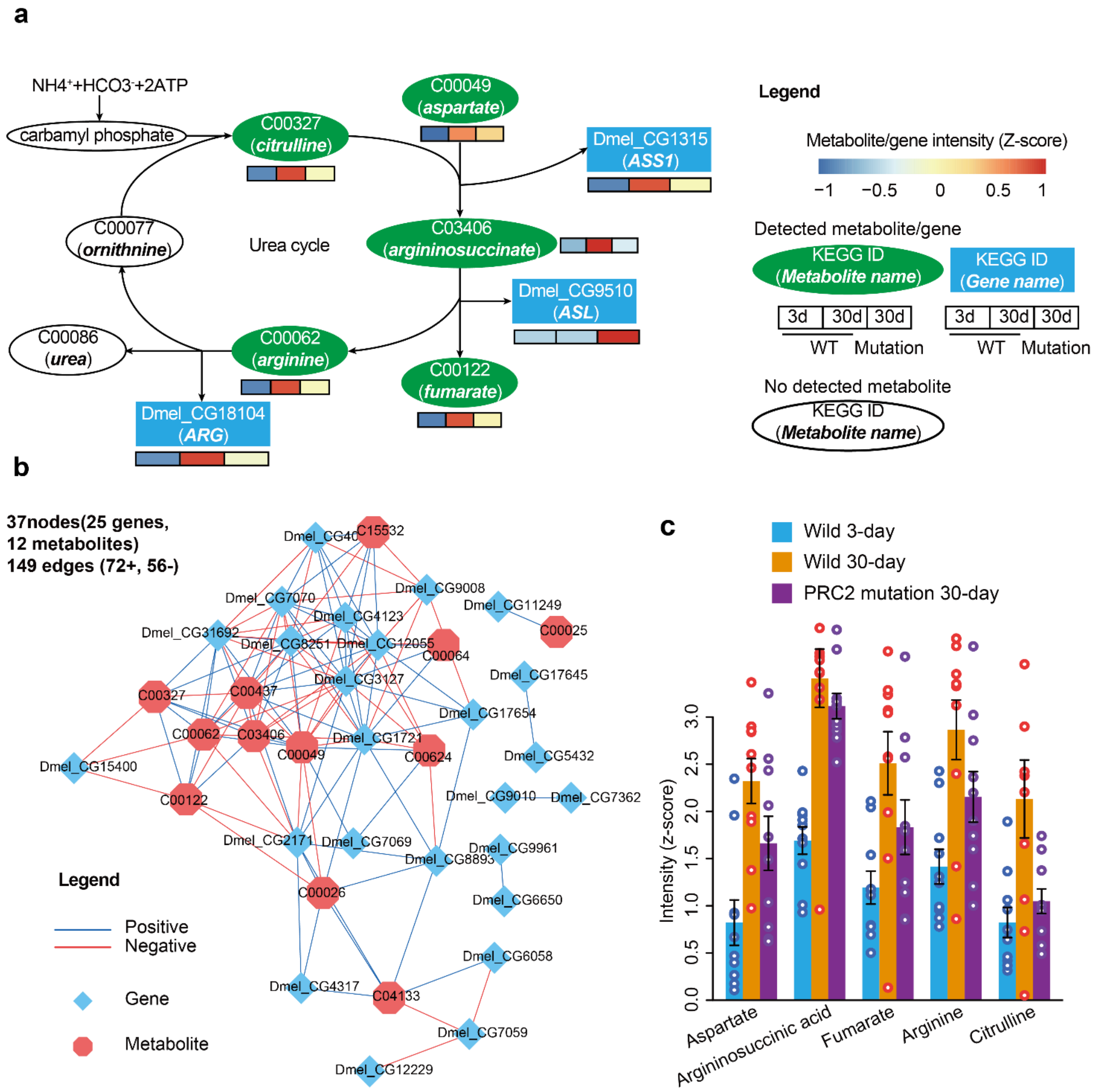

Supplementary Figure 21

The dysregulated arginine biosynthesis pathway in *Drosophila* aging datasets.

(**a**) Diagram illustrates that the transcriptional and metabolic changes in arginine biosynthesis pathway in 3d- and 30d-old WT and long-lived PRC2 mutant fruit flies. (**b**) The correlation network of genes and metabolites in the arginine biosynthesis pathway. One node represents one gene or one metabolite. The connections were established by Pearson correlation (Student's t-test, *P*-values < 0.05 and absolute Pearson correlation values > 0.7). (c) Barplots show five detected metabolites, Aspartate, Argininosuccinic acid, Fumarate, arginine and Citrulline in 3d- and 30d-old WT and long-lived PRC2 mutant fruit flies. The y-axis represents the z-score of metabolites (mean ± SD), n = 10 biologically independent samples for each group.

Supplementary Table 1

The detailed information of 28 metabolites confirmed using chemical standards, METLIN, NIST, HMDB library and CFM-ID.

Metabolite name	KEGG	Peak name	m/z error (ppm)	RT error	DP	Library
Arginyl-Alanine	C05340	M246T775	5.69	18.6s	0.98	Chemical standard
N-(omega)-Hydroxyarginine	C05933	M232T816	4.74	21.7s	0.98	Chemical standard
Argininosuccinic acid	C03406	M291T872	5.15	27.6s	0.99	Chemical standard
N-a-Acetyl-L-arginine	C01402	M217T681	1.38	15.6s	0.88	Chemical standard
DL-Arginine	C02385	M175T687	17.7	29.3s	0.98	Chemical standard
L-Aspartate	C00049	M134T728	8.95	30.3s	0.90	Chemical standard
L-Hydroxyarginine	C20330	M232T686	2.69	19.8%	0.97	METLIN
D-Ornithine	C00515	M175T961	2.64	28.07%	0.96	METLIN
4-Guanidinobutanoate	C01035	M128T657	20.1	3.4%	0.6	METLIN
D-Arginine	C00792	M175T961	12.08	8.6%	0.99	NIST
N2-Succinyl-L-ornithine	C03415	M255T677	3.35	8.67%	0.57	HMDB
N-Succinyl-L-glutamate 5-semialdehyde	C05932	M249T766	20.28	10.20%	0.51	HMDB
4-Diaminopentanoate	C03943	M196T604	2.64	34.5%	0.96	CFM-ID
Deoxyguanidinoproclavaminic acid	C06656	M246T775	3.49	16.1%	0.72	CFM-ID
N2-Succinyl-L-arginine	C03296	M232T554_1	10.32	10.2%	0.66	CFM-ID
L-Capreomycidine	C18472	M214T600	2.11	5.27%	0.63	CFM-ID
4-Guanidinobutanamide	C03078	M209T598	2.7	17.1%	0.61	CFM-ID
beta-Hydroxyarginine	C18473	M257T609	19.37	28.5%	0.51	CFM-ID
N2-(2-Carboxyethyl)-L-arginine	C06655	M247T645	3	23.5%	0.4	CFM-ID
Guanidinoproclavaminic acid	C06657	M245T554	3.71	40.15%	0.4	CFM-ID
4-Guanidinobutanal	C02647	M130T468	1.75	10.81%	0.37	CFM-ID
L-pHPg-L-Arg	C20910	M306T670	0.52	27.5%	0.35	CFM-ID
L-4-Hydroxyphenylglycine	C12323	M209T598	20.78	8.9%	0.35	CFM-ID

Proclavaminic acid	C06658	M203T657	3.14	6.49%	0.26	CFM-ID
5-Guanidino-2-oxopentanoate	C03711	M174T663	2.13	8.6%	0.25	CFM-ID
Iminoarginine	C21026	M216T601	7.13	31.84%	0.22	CFM-ID
5-Guanidino-3-methyl-2-oxopentanoate	C20234	M232T554_1	10.32	10.2%	0.15	CFM-ID
N-Succinyl-L-citrulline	C18048	M314T799	9.75	2.12%	0.1	CFM-ID

Supplementary Table 2

The detailed information of all the datasets used to evaluate the performance of MetDNA in this study.

Dataset ID	Species	Sample type	Sample group	Column type (Gradient time)	Data acquisition	Instrument platform
**1**	*Drosophila melanogaster* (fruit fly)	Brain tissue	Wild type (5905); 3-day *vs.* 30-day (n = 10 each)	Amide (23min)	DDA	Sciex TripleTOF 6600
**2**	*Mus musculus* (mouse)	Liver tissue	Mouse (c57BL/6J); 24-week *vs.*78-week (n = 10 each)	Amide (23min)	MS1+MS2 (QC)	Sciex TripleTOF 6600
**3**					SWATH	Sciex TripleTOF 6600
**4**					DDA	Thermo Q-Exactive HF-X
**5**		Cell	MEF cell; WT *vs.* $RIP1^{-/-}$ (n = 6 each)	Amide (12min)	DDA	Sciex TripleTOF 6600
**6**				C18 (12min)	DDA	Sciex TripleTOF 5600
**7**	*Caenorhabditis elegans* (nematode)	Whole body tissue	Worm WT *vs.* daf-2 mutant (n = 6 each)	Amide (23min)	DDA	Sciex TripleTOF 6600
**8**				Amide (23min)	MS1+MS2(QC)	Agilent QTOF 6550
**9**	*Escherichia coli* (*E. coli*)	Cell	*E. coli* (BL21DE3) WT *vs.* WT with protein expression (n = 10 each)	Amide (23min)	DDA	Sciex TripleTOF 6600
**10**	*Homo sapiens* (human)	Colorectal tissue	Colorectal cancer patients； Tumor *vs.* adjacent normal tissues (n = 10 pairs)	Amide (23min)	SWATH	Sciex TripleTOF 6600
**11**		Urine	Normal *vs.* Esophagus cancer patients (n = 20 each)	ZIC-HILIC (20min)	DDA	Sciex TripleTOF 5600

Supplementary Table 2 continued

Dataset ID	Data Availability
**1**	http://www.zhulab.cn/uploads/file/MetDNA/dataset/Dataset1.zip
**2**	http://www.zhulab.cn/uploads/file/MetDNA/dataset/Dataset2.zip
**3**	http://www.zhulab.cn/uploads/file/MetDNA/dataset/Dataset3.zip
**4**	http://www.zhulab.cn/uploads/file/MetDNA/dataset/Dataset4.zip
**5**	http://www.zhulab.cn/uploads/file/MetDNA/dataset/Dataset5.zip
**6**	http://www.zhulab.cn/uploads/file/MetDNA/dataset/Dataset6.zip
**7**	http://www.zhulab.cn/uploads/file/MetDNA/dataset/Dataset7.zip
**8**	http://www.zhulab.cn/uploads/file/MetDNA/dataset/Dataset8.zip
**9**	http://www.zhulab.cn/uploads/file/MetDNA/dataset/Dataset9.zip
**10**	http://www.zhulab.cn/uploads/file/MetDNA/dataset/Dataset10.zip
**11**	http://www.zhulab.cn/uploads/file/MetDNA/dataset/Dataset11.zip

Supplementary Table 3

Metabolite annotation and pathway enrichment analysis results of all the datasets in this study.

Dataset ID	Species	Sample type	Detected peaks (Positive/Negative)	Metabolite (Positive/Negative/In total)	Dysregulated peaks	Enriched pathways
**1**	*Drosophila melanogaster* (fruit fly)	Brain tissue	18,320/14,965	1,314/1,402/1,983	917[a]	25
**2**	*Mus musculus* (mouse)	Liver tissue	21,607/18,091	1,301/1,373/1,901	1,294[b]	16
**3**			14,532/10,914	1,699/1,550/2,296	711[b]	21
**4**			12,507/17,025	516/658/953	569[b]	27
**5**		Cell	27,270/20,615	1,390/1,288/1,976	896[c]	22
**6**			12,783/11,781	336/263/519	278[c]	16
**7**	*Caenorhabditis elegans* (nematode)	Whole body tissue	45,370/45,310	2,013/2,069/2,747	4,143[c]	28
**8**			35,992/35,178	407/632/837	1,382[c]	30
**9**	*Escherichia coli* (*E. coli*)	Cell	35,498/22,534	1,713/1,590/2,340	2,496[c]	36
**10**	*Homo sapiens* (human)	Colorectal tissue	8,797/4,467	1,334/1,087/1,893	304[c]	23
**11**		Urine	15,721/16,891	1,451/1,854/2,355	829[b]	22

a: Student's *t*-test, FDR-corrected *P* values < 0.01; b: Student's *t*-test, *P*-values < 0.05; c: Student's *t*-test, FDR-corrected *P* values < 0.05.

Supplementary Table 4

Correct and four types of misannotated seed metabolites in positive mode of *Drosophila* aging dataset (dataset #1).

Correct and four types of misannotated seed metabolites were constructed based on m/z error, RT error and MS2 spectral similarity. And then both correct and misannotated seed metabolites gone through the MRN based recursive annotation.

	Correct seed metabolite	Wrong seed metabolite			
		Type 1	Type 2	Type 3	Type 4
**m/z error**	< 25 ppm	> 25 ppm	< 25 ppm	< 25 ppm	< 25 ppm
**RT error**	< 60 s	> 60 s	> 60 s	< 60 s	> 60 s
**Dot product**	> 0.8	-	-	-	> 0.8
**Peak number**	167	167	167	164	164
**Metabolite number**	107	169	130	144	116
**Edge/node density (mean ± SEM)**	8.2±2.8	10.8±2.9	10.3±3.9	7.5±3.6	6.1±2.7
**Number of annotated peaks (Removing seeds)**	2,106	41	183	213	262
**Number of annotated metabolites (Removing seeds)**	1,282	34	143	160	218
**Round of annotation**	21	4	9	6	12

Supplementary Table 5

Adduct ion table used in MetDNA.

Adduct	Mass difference	Ionization polarity	Liquid chromatograph
$[2M+H]^+$	1.0073	Positive	HILIC;RP
$[2M+K]^+$	38.9632	Positive	HILIC;RP
$[2M+Na]^+$	22.9893	Positive	HILIC;RP
$[2M+NH4]^+$	18.0339	Positive	HILIC;RP
$[M-2H+3K]^+$	114.875	Positive	HILIC;RP
$[M-2H+3Na]^+$	66.9533	Positive	HILIC;RP
$[M-H+2K]^+$	76.9191	Positive	HILIC;RP
$[M-H+2Na]^+$	44.9713	Positive	HILIC;RP
$[M+2K-H]^+$	76.91904	Positive	HILIC
$[M+2Na-H]^+$	44.97116	Positive	HILIC
$[M+3Na-2H]^+$	66.9538	Positive	HILIC
$[M+CH_3CN+H]^+$	42.0338	Positive	HILIC;RP
$[M+CH_3CN+Na]^+$	64.0158	Positive	HILIC;RP
$[M+CH_3COO+2H]^+$	61.0284	Positive	HILIC
$[M+H-2H_2O]^+$	-35.0139	Positive	HILIC;RP
$[M+H-H_2O]^+$	-17.0033	Positive	HILIC;RP
$[M+H]^+$	1.0073	Positive	HILIC;RP
$[M+H+HCOOH]^+$	47.012746	Positive	RP
$[M+HCOO+2H]^+$	47.0128	Positive	RP
$[M+K]^+$	38.9632	Positive	HILIC;RP
$[M+K+HCOOH]^+$	84.968628	Positive	RP
$[M+Na]^+$	22.9893	Positive	HILIC;RP
$[M+Na+HCOOH]^+$	68.994688	Positive	RP
$[M+NH_4]^+$	18.0344	Positive	HILIC;RP
$[2M-H]^-$	-1.0073	Negative	HILIC;RP
$[M-2H]^{2-}$	-2.014552	Negative	HILIC;RP
$[M-2H+K]^-$	36.948606	Negative	HILIC;RP
$[M-2H+Na]^-$	20.974666	Negative	HILIC;RP
$[M-H-H_2O]^-$	-19.022576	Negative	HILIC;RP
$[M-H]^-$	-1.0073	Negative	HILIC;RP
$[M-H+CH_3CN]^-$	40.01872	Negative	HILIC;RP
$[M-H+NH_3]^-$	16.019274	Negative	HILIC;RP
$[M-H_2O-H]^-$	-19.0179	Negative	HILIC
$[M+CH_3COO]^-$	59.0138	Negative	HILIC
$[M+Cl]^-$	34.969402	Negative	HILIC;RP
$[M+Cl+NH_3]^-$	51.995952	Negative	HILIC;RP
$[M+F]^-$	18.9989	Negative	RP
$[M+K-2H]^-$	36.9486	Negative	HILIC;RP
$[M+Na-2H]^-$	20.9747	Negative	HILIC;RP
$[M+NH_4-2H]^-$	16.0193	Negative	HILIC;RP

Supplementary Table 6

The detailed parameters for data conversion using ProteoWizard.

Parameters	mzXML conversion	mgf conversion
**Output format**	mzXML	mgf
**Binary encoding precis**	64-bit	64-bit
**Write index**	YES	YES
**Use zlib compression**	YES	YES
**TPP compatibility**	YES	YES
**Package in gzip**	NO	NO
**Use numpress linear compression**	NO	NO
**Use numpress short logged floate compression**	NO	NO
**Use numpress short positive inter compression**	NO	NO
**Filters**	Peak Picking	Peak Picking

Supplementary Note 1

Our in-house library (841 metabolites), NIST17 library [https://chemdata.nist.gov/] and METLIN library [https://metlin.scripps.edu/] were used. A total of 542, 220 and 248 reaction pairs were retrieved from our in-house spectral library, the NIST17 library and METLIN library, respectively. Meanwhile, the same numbers of non-reaction pairs were also randomly generated for the comparison. Specifically, for each metabolite, its neighbor metabolites in library (in-house, NIST17 or METLIN) were retrieved and utilized to construct the reaction pairs (RPs). Meanwhile, the metabolites in the library and have the smallest m/z error with the true neighbor metabolites were also retrieved and utilized to construct non-RPs. For example, if 'metabolite a' has a neighbor metabolite 'b' in the library, so 'a-b' is constructed as a RP. And then the 'metabolite c' with the smallest m/z error with 'metabolite b' in the library is used to construct the non-RP. The spectral similarity was scored using the dot-product function (DP score, see Methods). In our in-house library, more than 55.3% of neighbor metabolites have a DP score larger than 0.5. In contrast, only 5.2% of non-neighbor metabolites have a DP score larger than 0.5. In NIST library, more than 53.6% of neighbor metabolites have a DP score larger than 0.5. In contrast, only 3.6% of non-neighbor metabolites have a DP score larger than 0.5. In METLIN library, more than 53.4% of neighbor metabolites have a DP score larger than 0.5. In contrast, only 3.2% of non-neighbor metabolites have a DP score larger than 0.5. We further explored the appropriate cutoff of DPs. When the cutoff is set as 0.8, the percentage of non-reaction pairs with DPs larger than 0.8 decreases to 2.0%. However, the percentage of reaction pairs with DPs larger than 0.8 significantly decreases to 37.3%. So we think the cut-off score of 0.5 is appropriate.

To demonstrate how the cosine similarities decay as a function of the reaction 'distance' between two compounds, we constructed RPs and non-RPs with 2, 3, 4 and 5 steps, respectively. As shown in Supplementary Fig. 1b, the percentages of RPs with DP > 0.5 significantly decreased to 26.5%, 15.5%, 10.3% and 8.0% from 55.3% (with 1 step) for 2, 3, 4 and 5 reaction steps, respectively.

We designed an experiment described as follows to compare the bonanza score[1], Hybrid Similarity Search (HSS) score[2], GNPS[3] and dot product (DP) score. In brief, RPs retrieved from our in-house library were utilized to calculated DP, bonanza, HSS and GNPS scores, respectively. As shown in Supplementary Fig. 1c, most of bonanza scores were smaller than dot products, especially for the RPs with high DP scores. However, the DP and bonanza scores from ~74.0% of RPs had no significant differences (absolute difference < 0.2). In addition, the percentage of RPs with bonanza scores larger than 0.5 decreased to 30.9% compared to

55.3% using DP. Meanwhile, the percentage of non-RPs with bonanza scores larger than 0.5 decreased to 2.0% compared with 5.2% using DP score (Supplementary Fig. 1f). On the contrary, most of HSS scores were larger than DP scores, especially for the RPs with low DP scores (Supplementary Fig. 1d). However, the DP and HSS scores from ~80.0% of RPs had no significant differences (absolute difference < 0.2). In addition, the percentage of RPs with HSS scores larger than 0.5 increased to 63.9% compared with 55.3% using DP score. Meanwhile, the percentage of Non-RPs with HSS scores larger than 0.5 also increased to 10.2% compared to 5.2% using DP score (Supplementary Fig. 1f). As noted, all GNPS scores were equal to or greater than DP scores (Supplementary Fig. 1e). However, the DP and GNPS scores from ~87.0% of RPs had no significant differences (absolute difference < 0.2). In addition, the percentage of RPs with GNPS scores larger than 0.5 increased to 67.3% compared with 55.3% using DP score. Meanwhile, the percentage of Non-RPs with GNPS scores larger than 0.5 also increased to 10.4% compared to 5.2% using DP score (Supplementary Fig. 1f).

Since bonanza score is a more strict scoring system than DP score. Presumably, the use of bonanza score may decrease the false positives in MetDNA, but it may also increase the false negatives and decrease the number of annotated metabolites in the same time. On the contrary, the use of HSS score and GNPS may have an inverse effect (*i.e.*, lower false negatives but higher false positive rate). If one would incorporate the bonanza, HSS or GNPS score into MetDNA, the systematic optimization of score cut-off and evaluation of the validation results are required. Therefore, we think dot product score is appropriate enough in the MetDNA.

Supplementary Note 2

(1) Optimization of RT match threshold

To systemically optimize the retention time match tolerance, two experiments were designed.

Experiment 1

For positive mode of *Drosophila* aging dataset, the metabolite annotation was first performed using our in-house standard tandem spectral library through m/z (m/z match error < 25 ppm) and MS spectral match (Dot product > 0.8). Then the peaks with annotations were confirmed using measured standard RTs (RT match error < 60 s), and labeled as Annotation 1. Meanwhile, these peaks with annotations were also matched with the theoretical RTs using different match tolerances, and labeled as Annotation 2. The RT match threshold was set from 10% to 100% with a step 10%, and infinity (i.e., removing the RT match). Then the Annotation 2 was compared to Annotation 1 to calculate the true positive rate and true negative rate according to the rule in Supplementary Table 7. According to the distributions of true positive and true negative rates, a RT match tolerance of 30% is determined (Supplementary Fig. 7).

Supplementary Table 7

Rules for calculating true positive and true negative rates in validation experiment 1.

Annotation 1	Annotation 2	Note
A	A	True positive
Not B	Not B	True positive
C	D	False positive
E	NA	False negative
NA	F	False positive

Experiment 2

For positive mode of *Drosophila* aging dataset, the initial seed metabolites were selected according to the workflow of MetDNA. Then 30% of initial seed metabolites were randomly selected as initial seed metabolites for MRN-based recursive metabolite annotation, and the remained 70% were as the validation dataset (similar to Validation experiment #2). This process was repeated 10 times. In order to evaluate the influence of RT match threshold on the annotation, the RT match threshold was set from 10% to 100% with a step 10%, and infinity (i.e., removing the RT match). For each RT match threshold, we ran the process described above. Finally, the peak redundancy and annotation correct rate using top 3 candidates were

calculated using the comparison between the validation dataset and annotations from MetDNA. The influences of RT match threshold on annotation correction rate and the peak redundancy were summarized in Supplementary Fig. 7b and 7c.

(2) Optimization of weights of annotation score

Five weights, $W_{m/z1}$ and W_{RT1} for $Score_{adduct}$ and $W_{m/z2}$, W_{RT2} and W_{spec} for $Score_{iden}$ were systemically optimized in MetDNA. We first defined the combinations of these weights as follows.

$$\begin{cases} W_{m/z1} = 0.1 + 0.05 * n \\ W_{RT1} = 0.1 + 0.05 * n \\ W_{m/z1} + W_{RT1} = 1 \\ n \in (0, 1, 2\ldots16) \end{cases}$$

$$\begin{cases} W_{m/z2} = 0.1 + 0.05 * n \\ W_{RT2} = 0.1 + 0.05 * n \\ W_{spec} = 0.1 + 0.05 * n \\ W_{m/z2} + W_{RT2} + W_{spec} = 1 \\ n \in (0, 1, 2\ldots16) \end{cases}$$

Finally, a total of 2,040 weight combinations were created, and used for MetDNA analysis one by one. A strategy similar in experiment 2 for optimizing RT match threshold was used. For each experiment, 30% of initial seed metabolites were randomly selected as initial seed metabolites for MRN-based recursive metabolite annotation, and the remained 70% were as the validation dataset. This process was repeated 10 times. In each experiment, a set of weight combination was used. Then, the annotation correction rate using top 3 candidates (no score cutoff) was calculated using the comparison between the validation dataset and annotations from MetDNA. Thus, a total of 2,040 annotation correction rates were calculated and plotted in Supplementary Fig. 8a. The correction rates ranged from 79.0% to 83.5%.

The weight combinations with correct rate > 82.0% (426 out of 2,040) were retrieved to investigate the optimal weight combinations. The value distributions of $W_{m/z1}$ and W_{RT1} in adduct annotation retrieved from the 426 combinations were plotted in Supplementary Fig. 8b. Clearly, the m/z match weight is generally higher than RT match weight. To simply the optimization process, four weigh combinations for adduction annotation were selected: 1) $W_{m/z1}$ is 0.9 and W_{RT1} is 0.1; 2) $W_{m/z1}$ is 0.85 and W_{RT1} is 0.15; 3) $W_{m/z1}$ is 0.8 and W_{RT1} is 0.2; and 4) $W_{m/z1}$ is 0.75 and W_{RT1} is 0.25. We further calculated the annotation coverages and correction rates using 12 weight combinations and different cutoffs of annotation scores (Supplementary Fig.

9). There were no significant differences in annotation coverage rates and correct rates when score cutoff was set from 0 to 0.4. When the score tolerance was set larger than 0.4, both annotation coverage and correction rate decreased (Supplementary Fig. 9). Therefore, the final score cutoff was determined as 0.4. For MetDNA, we recommend that the default weight combination was set as: $W_{m/z1}$, 0.8; W_{RT2}, 0.2; $W_{m/z2}$, 0.25; W_{RT2}, 0.25; and W_{spec}, 0.5.

Supplementary Note 3

In this study, a total of 11 datasets were used to evaluate the performance of MetDNA. These datasets cover five biological species (*D. melanogaster*, *M. musculus*, *C. elegans*, *E. coli*, and *H. sapiens*) and seven sample types (prokaryotic cells, whole body tissue, mammalian cells, brain tissue, liver tissue, colorectal tissue, and urine). Three types of liquid chromatography (HILIC: Amide and ZIC-HILIC columns; reverse phase: C18 column), four MS platforms (Agilent QTOF 6550, Sciex TripleTOF 5600 and 6600, Thermo Orbitrap Q-Extractive HF-X) and three different acquisition methods (data dependent acquisition (DDA), data independent acquisition (SWATH), and targeted MS2 acquisition) are also included (Supplementary Tables 2 and 3).

The information for dataset #1 (*Drosophila* aging dataset) was provided in Methods. Here, we provide more information for dataset #2-11.

Reagents

LC-MS water (H_2O), methanol (MeOH), 0.1% Formic acid (FA) in water, and 0.1% Formic acid (FA) in acetonitrile were purchased from Honeywell (Muskegon, USA). Acetonitrile (ACN) was purchased from Merck (Darmstadt, Germany). Ethanol was purchased from Sinopharm (Beijing, China). Ammonium hydroxide (NH_4OH) and ammonium acetate (NH_4OAc) were purchased from Sigma-Aldrich (St. Louis, USA). Metabolite chemical standards were purchased from J&K (Beijing, China), Sigma (St. Louis, USA), Carbosynth (Berkshire, UK), TCI (Tokyo, Japan) and Energy Chemical (Shanghai, China).

Study 1 (Datasets #2-4). Metabolism regulation of aging in mouse liver tissue

Two groups of aging mouse liver tissues (c57BL/6J; 24-week *vs.* 78-week; n = 10 for each group) were collected to study the dysregulated metabolic pathways in aging mouse.

Sample preparation

Mouse liver tissue samples (~10 mg) were first homogenized with ceramic beads and 200 μL of H_2O for three times using a Precellys homogenizers. The homogenization took 20 s with 5 s intervals for each time. Liquid nitrogen was used to keep the low temperature of homogenization. After homogenization, a volume of 400 μL of MeOH:ACN (1:1, v/v) was added to the samples as extraction solvent, vortexed for 30 s, and incubated in liquid nitrogen for 1 min. The samples were then allowed to thaw at room temperature, and sonicated for 10 min. This vortex-freeze-thaw cycle was repeated three times. To precipitate proteins, the

samples were incubated at -20 °C for 1 h, followed by centrifugation at 16,200 xg and 4 °C for 15 min. Then the supernatant was removed and evaporated to dryness in a vacuum concentrator. The dry extracts were then reconstituted in 100 μL of ACN:H_2O (1:1, v/v), then sonicated for 10 min, and centrifuged at 16,200 xg and 4 °C for 15 min to remove insoluble debris. The supernatant was then transferred to HPLC vials and stored at -80 °C prior to LC-MS analysis.

LC-MS analysis and data processing

Metabolomics data of aging mouse liver samples were acquired using different MS platforms and data acquisition methods (datasets #2-4 in Supplementary Table 2).

For dataset #2, metabolomics data acquisition was performed using a UHPLC system (1290 series, Agilent Technologies, USA) coupled to a quadruple time-of-flight mass spectrometer (TripleTOF 6600, AB SCIEX, USA). Waters ACQUITY UPLC BEH Amide column (particle size, 1.7 μm; 100 mm (length) × 2.1 mm (i.d.)) was used for the LC separation. The column temperature was kept at 25 °C. The mobile phase A was 25 mM ammonium hydroxide (NH_4OH) + 25 mM ammonium acetate (NH_4OAc) in water and B was ACN in both positive mode (ESI+) and negative mode (ESI-). The flow rate was 0.3 mL/min and the linear gradient was set as follows: 0-1 min: 95% B, 1-14 min: 95% B to 65% B, 14-16 min: 65% B to 40% B, 16-18 min: 40% B, 18-18.1 min: 40% B to 95% B, and 18.1-23 min: 95% B. The injection volume is 2 μL.

The metabolomics data acquisition mode was operated under information-dependent acquisition (IDA) mode. The source parameters were set as follows: ion source gas 1 (GAS1), 60 psi; ion source gas 2 (GAS2): 60 psi; curtain gas (CUR): 30 psi; temperature (TEM): 600 °C; declustering potential (DP): 60 V or -60 V in positive or negative modes, respectively; ion spray voltage floating (ISVF): 5500 V or -4000 V in positive or negative modes, respectively. The TOF MS scan parameters were set as follows: mass range: 60-1200 Da; accumulation time: 200 ms; dynamic background subtract: check. The product ion scan parameters were set as follows: mass range: 25-1200 Da, accumulation time: 50 ms; collision energy (CE): 30 V or -30 V in positive or negative mode, respectively; collision energy spread (CES): 0; resolution: UNIT; charge state: 1 to 1; intensity: 100 cps; exclude isotopes within: 4 Da; mass tolerance: 10 ppm; maximum number of candidate ions to monitor per cycle: 6; exclude former target ions: for 4 seconds after 2 occurrences. To expand the coverage of MS2 spectra, the mass range for precursor ions was divided into 3 segments, 50-300 Da, 290-600 Da and 590-1200 Da for QC samples.

For dataset #3, the SWATH technique on TripleTOF 6600 was used for data acquisition. The LC condition was same as the acquisition of dataset #2. The injection volume is 2 μL. For the SWATH setting, the

cycle time was set as 1 s, including one TOF MS scan (100 ms/scan; mass range: 60-1200 Da) and 24 SWATH-MS2 scans (35 ms/scan; 47.5 Da/SWATH window; mass range: 60-1200 Da). For MS2 acquisition, collision energy (CE): 30 V or -30 V in positive or negative mode, respectively; charge state: 1 to 1. All other MS parameters were the same as dataset #2.

For dataset #4, metabolomics data acquisition was performed using a UHPLC system (UltiMate3000 series, Thermo Fisher Scientific, USA) coupled to an Orbitrap mass spectrometer (Q-Exactive HF-X, Thermo Fisher Scientific, Germany). The mobile phase, gradient and column were the same as dataset #2. The injection volume is 2 μL. Spray voltage was set to 3500 V or -2800 V for positive or negative mode, respectively. Aux gas heater temperature was set as 350 °C. Sheath gas was set as 50 arb. Aux gas was set as 15 arb. Capillary temperature was set as 320 °C. Instrument was operated under data-dependent acquisition (DDA) mode using the full MS/dd-MS^2 setting. The full MS resolution was set as 60,000 and the AGC target was 1e6 or 3e6 for positive or negative mode, respectively. Maximum IT was set as 100 ms. Mass range was set to 70-1050 Da. For the dd-MS^2 settings, MS resolution was set as 15,000 and AGC target was set at 1e5. Maximum IT was set as 25 ms. The TopN setting was set as 6. Isolation width was set at 1.0 m/z. The fixed first mass setting was set as 50.0 m/z. Collision energy was set at 20 V or -20 V for positive or negative mode, respectively. In dd setting, the minimum AGC target was set as 5e3. The apex trigger was set as 2 to 8 s. The charge exclusion was set as 2-8, >8. Peptide match was set to off, and isotope exclusion was on. The dynamic exclusion was set as 3.0 s.

Datasets #2 and #4 were processed using R package xcms (version 1.46.0,) for peak detection and alignment. Then, the MS1 peak table from XCMS[4] and MS2 data (.mgf format converted using ProteoWizard [http://proteowizard.sourceforge.net/], version 3.0.6150) were processed using MetDNA. Dataset #3 was processed using MS-DIAL[5] (version 2.56). Then the MS1 peak table and MS2 data (.msp format) from MS-DIAL were also processed using MetDNA.

MetDNA analysis results

In the dataset #2, a total of 21,607 and 18,091 peaks were detected in positive and negative modes, respectively. After MetDNA annotation, a total of 1,901 metabolites were annotated (1,301, 1,373 and 773 for positive, negative and both modes, respectively). A total of 16 dysregulated metabolic pathways were enriched (Hypergeometric test, *P*-values < 0.05).

In the dataset #3, a total of 14,532 and 10,904 peaks were detected in positive and negative modes, respectively. After MetDNA annotation, a total of 2,296 metabolites were annotated (1,699, 1,550 and 953 for

positive, negative and both modes, respectively). A total of 21 dysregulated metabolic pathways were enriched (Hypergeometric test, *P*-values < 0.05).

In the dataset #4, a total of 12,507 and 17,025 peaks were detected in positive and negative modes, respectively. After MetDNA annotation, a total of 953 metabolites were annotated (516, 658 and 221 for positive, negative and both modes, respectively). A total of 27 dysregulated metabolic pathways were enriched (Hypergeometric test, *P*-values < 0.05).

Study 2 (Datasets #5 and #6). The metabolic regulation function of RIP1 in MEF cell

Two groups of MEF cells (wild-type and $RIP1^{-/-}$; n = 6 for each group) were used to study the dysregulated metabolic pathways in $RIP1^{-/-}$ cells.

Cell culture

Mouse embryonic fibroblast (MEF) cells (wild-type and $R1P1^{-/-}$) were obtained from Prof. Junying Yuan's Lab (Chinese Academy of Sciences, Shanghai) and cultured at 37 °C in 5% CO_2 in Dulbecco's Modified Eagle's Medium (DMEM) supplemented with 1% L-glutamine, 1% sodium pyruvate, 1% penicillin/streptomycin, and 10% FBS. The culture medium was replaced every 48 h to 72 h. The cells were collected for metabolite extraction before the confluency reached 60%.

Sample preparation

The MEF cell pellets (about 10^6 cells) were extracted using a MeOH:ACN:H_2O (2:2:1, v/v) solvent mixture. A volume of 1 mL of cold solvent was added to each cell pellet, vortexed for 30 s and incubated in liquid nitrogen for 1 min. The samples were then allowed to thaw at room temperature and sonicated for 10 min. This freeze–thaw cycle was repeated three times. Then the samples were incubated for 1 h at -20 °C, followed by 15 min centrifugation at 16,200 xg and 4 °C to precipitate proteins. The resulting supernatant was removed and evaporated to dryness in a vacuum concentrator. The dry extracts were then reconstituted in 100 μL of ACN:H_2O (1:1, v/v), sonicated for 10 min, and centrifuged 15 min at 16,200 xg and 4 °C to remove insoluble debris. The supernatants were transferred to HPLC vials and stored at -80 °C prior to LC/MS analysis.

LC-MS analysis and data processing

For dataset #5, metabolomics data acquisition was performed similar to dataset #2, expect a 12-min gradient was used. The flow rate was 0.5 mL/min and the linear gradient was set as follows: 0-0.5 min: 95% B, 0.5-7 min: 95% B to 65% B, 7-8 min: 65% B to 40% B, 8-9 min: 40% B, 9-9.1 min: 40% B to 95% B, and

9.1-12 min: 95% B. The injection volume is 2 μL.

For dataset #6, metabolomics data acquisition was performed using a UHPLC system (1290 series, Agilent Technologies, USA) coupled to a quadruple time-of-flight mass spectrometer (TripleTOF 5600, AB SCIEX, USA). Waters ACQUITY UPLC HSS T3 columns (particle size, 1.8 μm; 100 mm (length) × 2.1 mm (i.d.)) were used for the LC separation. The column temperature was kept as 25 °C. The flow rate was 0.5 mL/min. The mobile phases A was 0.1% formic acid in water in positive mode (ESI+) or 5 mM NH_4Ac in water in negative mode (ESI-), and B was 0.1% formic acid in ACN in positive mode or 100% ACN in negative mode, respectively. The linear gradient was set as follows: 0-1 min: 1% B, 1-8 min: 1% B to 100% B, 8-10 min: 100% B, 10-10.1 min: 100% B to 1% B, and 10.1-12 min: 1% B. The injection volume is 6 μL.

The metabolomics data acquisition mode was operated under data-dependent acquisition (DDA) mode. The source parameters were set as follows: ion source gas 1 (GAS1), 60 psi; ion source gas 2 (GAS2): 60 psi; curtain gas (CUR): 35 psi; temperature (TEM): 650 °C; declustering potential (DP): 60 V or -60 V in positive or negative modes, respectively; ion spray voltage floating (ISVF): 5000 V or -4000 V in positive or negative modes, respectively. The TOF MS scan parameters were set as follows: mass range: 60-1200 Da; accumulation time: 200 ms; dynamic background subtract: check. The product ion scan parameters were set as follows: mass range: 25-1200 Da, accumulation time: 50 ms; collision energy (CE): 30 V or -30 V in positive or negative mode, respectively; collision energy spread (CES): 0; resolution: UNIT; charge state: 1 to 1; intensity: 100 cps; mass tolerance: 10 ppm; maximum number of candidate ions to monitor per cycle: 6; exclude former target ions: for 4 seconds after 2 occurrences. To expand the coverage of MS2 spectra, the mass range for precursor ions was divided into 3 segments, 50-300 Da, 290-600 Da and 590-1200 Da for QC samples.

Datasets #5 and #6 were processed using R package xcms[4] and MetDNA.

MetDNA analysis results

In the dataset #5, a total of 27,270 and 20,615 peaks were detected in positive and negative modes, respectively. A total of 1,976 metabolites were annotated (1,390, 1,288 and 702 for positive, negative and both modes, respectively). A total of 22 dysregulated metabolic pathways were enriched (Hypergeometric test, P-values < 0.05).

In the dataset #6, a total of 12,783 and 11,781 peaks were detected in positive and negative modes, respectively. A total of 519 metabolites were annotated (336, 263 and 80 for positive, negative and both modes, respectively). A total of 16 dysregulated metabolic pathways were enriched (Hypergeometric test, P-values <

0.05).

Study 3 (Datasets #7 and #8). Metabolic changes between wide-type and daf-2 mutant *C. elegans*

Two groups of *C. elegans* (wild-type *vs.* daf-2 mutant, n = 6 for each group) were used to study the dysregulated metabolic pathways in daf-2 mutated *C. elegans*.

Sample preparation

C. elegans samples were collected into microcentrifuge tubes and then homogenized with ceramic beads and 200 μL of H_2O for three times using Precellys homogenizers. The rest metabolite extraction was the same as the extraction of mouse liver tissues.

LC-MS analysis and data processing

For dataset #7, the data acquisition was performed the same as dataset #2.

For dataset #8, metabolomics data acquisition was performed using a UHPLC system (1290 series, Agilent Technologies, USA) coupled to a quadruple time-of-flight mass spectrometer (Agilent 6550 iFunnel Q-TOF, Agilent Technologies, USA). The mobile phase, column and linear gradient were the same as dataset #2. The injection volume is 2 μL. The parameters of MS data acquisition were set as follows: sheath gas temperature, 400 °C; dry gas temperature, 250 °C; sheath gas flow, 12 L/min; dry gas flow, 16 L/min; capillary voltage, 3000 V in positive mode or -3000 V in negative mode, respectively; nozzle voltage, 0 V; and nebulizer pressure, 20 psi in positive or 40 psi in negative mode, respectively. The MS acquisition rate was set as 4 spectra/s. The mass range was set as m/z 50-1200 Da. Samples are acquired using MS1 only mode. The MS2 data were acquired from the QC samples using the Auto MS/MS mode. To expand the coverage of MS2 spectra, the mass range for precursor ions was divided into 6 segments, 60-180 Da, 170-300 Da, 290-450 Da, 440-600 Da, 590-900 Da and 890-1200 Da for QC samples. The mass range was set as 50-1200 Da and 25-1200 Da for MS and MS/MS acquisitions, respectively. The acquisition rates were set as 5 and 2 spectra/s for MS and MS/MS acquisitions, respectively. The max precursor per cycle was set as 2. The collision energy was set as 20 V and -20V for positive and negative modes, respectively.

Dataset #7 and #8 were processed using R package xcms[4] and MetDNA.

MetDNA analysis results

In the dataset #7, a total of 45,370 and 45,310 peaks were detected in positive and negative modes, respectively. A total of 2,747 metabolites were annotated (2,013, 2,069 and 1,335 for positive, negative and both modes, respectively). A total of 28 dysregulated metabolic pathways were enriched (Hypergeometric test,

P-values < 0.05).

In the dataset #8, a total of 35,992 and 35,178 peaks were detected in positive and negative modes, respectively. A total of 837 metabolites were annotated (407, 632 and 202 for positive, negative and both modes, respectively). A total of 30 dysregulated metabolic pathways were enriched (Hypergeometric test, *P*-values < 0.05).

Study 4 (Dataset #9). Metabolomic profiling of *E. coli* with protein (α-syn) expression

Two groups of *E. coli* samples (wild-type *vs. E. coli* with α-syn expression, n = 10 for each group) were used to study the dysregulated metabolic pathways in *E. coli* with protein (α-syn) expression.

Sample preparation

The *E. coli* cell samples ($OD_{600\ nm}$ = 1.0, 10 mL) were extracted using a MeOH:ACN:H_2O (2:2:1, v/v) solvent mixture. The rest metabolite extraction was the same as the extraction of MEF cell sample.

LC-MS analysis and data processing

The LC-MS analysis and data processing of dataset #9 are the same as dataset #2.

MetDNA analysis results

In dataset #9, a total of 35,498 and 22,534 peaks were detected in positive and negative modes, respectively. A total of 2,340 metabolites were annotated (1,713, 1,590 and 963 for positive, negative and both modes, respectively). A total of 36 pathways dysregulated metabolic pathways were enriched (Hypergeometric test, *P*-values < 0.05).

Study 5 (Dataset #10). Metabolomic profiling of human colorectal cancer tissues

Two groups of colorectal tissues (cancer *vs.* adjacent healthy tissues, n = 10 for each group) were used to study the dysregulated metabolic pathways in colorectal cancer tissues.

Sample collection

The tissue samples of colorectal cancer patients were collected from the Department of Gynecology of Harbin Medical University Tumor Hospital (Harbin, Heilongjiang Province, China). The patients were enrolled with the written informed consents. The study was approved by the Ethics Committee of the Tumor Hospital of Harbin Medical University (Harbin, China). All the patients had undergone colorectal surgery and their diagnosis had been confirmed by histopathologic examinations. Tumors of CRC patients were staged according to the Union for International Cancer Control (UICC) pathologic tumor-node-metastasis (TNM)

classification system (8th edition, 2016). Detailed baseline and histopathologic characteristics for these patients are listed in Supplementary Table 8. The tumor tissues were excised from CRC patients during surgery. Meanwhile, the adjacent healthy tissues were surgically excised at 5 to 10 cm away from the tumor. The tissue samples were immediately frozen using liquid nitrogen and stored at -80 °C until metabolite extraction.

Supplementary Table 8

Baseline and histopathologic characteristics of participant subjects in Study 5.

	**Colorectal patient**
**Sample size**	10
**Age, mean (sd, year)**	63.7 (9.0)
**Gender Males, n (%)**	6 (60)
**BMI, mean (sd)**	22.2 (3.1)
**Smoker, n (%)**	6 (60)
**Drinker, n (%)**	2 (20)
**Tumor size, mean (sd, cm)**	4.2 (1.6)

Sample preparation

The sample preparation of colorectal tissues was the same as the preparation of mouse liver tissues.

LC-MS analysis and data processing

The LC-MS analysis and data processing of colorectal sample are the same as dataset #3.

MetDNA analysis results

In dataset #10, a total of 8,797 and 4,467 peaks were detected in positive and negative modes, respectively. A total of 1,893 metabolites were annotated (1,334, 1,087 and 528 for positive, negative and both modes, respectively). A total of 23 dysregulated metabolic pathways were enriched (Hypergeometric test, P-values < 0.05).

Study 6 (Dataset #11). Metabolomic profiling of urine samples from esophagus cancer patients

Two groups of urine samples from patients with esophagus cancer (EC) and healthy controls (n = 20 for each group) were collected to study the dysregulated metabolic pathways in patients with esophagus cancer.

Sample collection

Urine samples were collected at the Esophageal Cancer Screening Base of Shandong Province (Feicheng, Shandong Province, China). The study was approved by the Ethics Committee of the Shandong Tumor Hospital and written informed consent was obtained from all participants involved in this study. All the participants with ages 40-69 years enrolled in this base were screened for esophageal cancer using endoscopy with mucosal iodine staining. In this study, participants with normal esophageal mucosa (iodine-positive) was regarded as the health controls. Biopsies of the participants with iodine-negative pathology were taken from the non-staining area of the mucosa, which were then underwent pathological evaluation for confirming and staging by two independent pathologists. Each EC patient was diagnosed and staged according to the American Joint Committee on Cancer (AJCC) TNM Classification of Carcinoma of the Esophagus and Esophagogastric Junction (7th edition, 2010). Detailed baseline and histopathologic characteristics for these patients are listed in Supplementary Table 9. Participants involved in this study were not taking any medications, surgery, radiotherapy or chemotherapy, and those suffering from metabolic diseases, liver diseases, kidney diseases or any other cancers were excluded. All of the participants were in an overnight fasting state and 5 mL of urine was taken in the morning. The urine samples were stored at −80 °C until further analysis.

Supplementary Table 9

Baseline and histopathologic characteristics of participant subjects in Study 6.

	Healthy control	EC patient	*P*-value
**Sample size**	20	20	-
**Age, mean(sd, year)**	58.7 (6.3)	61.4 (6.4)	0.188[a]
**Gender Males, n (%)**	10 (50)	9 (45)	1[b]
**BMI, mean (sd)**	23.5 (2.4)	22.3 (2.3)	0.109[a]
**Smoker, n (%)**	6 (30)	4 (20)	0.715[b]
**Drinker, n (%)**	9 (45)	6 (30)	0.1769[b]

a, Student’s-*t* test; b, Chi-squared test.

Sample preparation

Urine samples were thawed at 4 °C on ice. Then, 50 μL of urine sample was taken and transferred into a microcentrifuge tube, and 150 μL of water was added to dilute urine. The diluted urine samples were centrifuged at 21,600 xg and 4 °C for 15 min. Finally, 100 μL of supernatant was then transferred to HPLC

glass vials and stored at -80 °C prior to LC-MS analysis.

LC-MS analysis and data processing

For dataset #11, metabolomics data acquisition was performed similar to dataset #2, expect for the mobile phase, column and linear gradient. A Merck SeQuant ZIC-HILIC column (particle size, 3.5 μm; 100 mm (length) × 2.1 mm (i.d.)) was used for the LC separation and the column temperature was kept at 25 °C. The mobile phase A was 10 mM ammonium acetate (NH_4OAc) in 95% water and 5% acetonitrile, and B was 10 mM ammonium acetate (NH_4OAc) in 5% water and 95% acetonitrile both in positive mode (ESI+) and negative mode (ESI-). The flow rate was 0.3 mL/min and the linear gradient was set as follows: 0-13 min: 99% B to 40% B, 13-15 min: 40% B, 15-15.1 min: 40% B to 99% B, and 15.1-20 min: 99% B. The injection volume is 2 μL.

The data processing methods are the same with dataset #2.

Metabolite annotation and pathway enrichment analysis results

For dataset #11, a total of 15,721 and 16,891 peaks were detected in positive and negative modes, respectively. A total of 2,355 metabolites were annotated (1,451, 1,854 and 950 for positive, negative and both modes, respectively). A total of 22 pathways were enriched (Hypergeometric test, *P*-values < 0.05).

Supplementary Note 4

Validation experiment 1

A total of 200 metabolite standards (Supplementary Table 4) were manually spiked into the mouse liver samples, and then analyzed by LC-MS/MS and processed using MetDNA (sample preparation, data acquisition and all data processing are same as dataset #4 in Supplementary Note 3). The 200 chemical standards were chosen from our in-house library with no bias, which were described in our recent publication[6]. A total of 167 chemical standards (83.5%) were included in metabolic reaction network (MRN). And the samples were acquired data using LC-MS/MS. Then, the data was processed using XCMS for peak detection and alignment, and the customized R script was used to extract MS2 spectra. Manual analysis of the experimental data with the comparison to the data of chemical standards (m/z, RT, and MS2 spectra) demonstrated that 113 and 137 metabolites were detected in positive and negative modes, respectively. The data of chemical standards was individually measured using the single chemical standard under the same LC-MS/MS condition. The match criteria were set as m/z error < 25 ppm, RT error < 30 s and DP > 0.8. According to MSI, the identifications were considered as the Level 1 identification.

As a comparison, the same LC-MS/MS data were also processed using MetDNA. MetDNA successfully annotated 89 (out of 113) and 113 (out of 137) metabolites in positive and negative modes, respectively. Therefore, we calculated the annotation rates as 78.8% (89/113) and 82.5% (113/137), respectively. We further evaluated the sensitivity and specificity thorough the calculation of true positive and false positive rates. To do so, the annotations from MetDNA were compared to the identification results from manual analysis, and divided into three categories: correct, isomeric and erroneous annotations.

Validation experiment 2

A validation strategy was designed to validate the metabolite annotations from MetDNA (Supplementary Fig. 14). First, the chemical structures of initial seed metabolites from positive mode of *Drosophila* aging dataset are confirmed using metabolite standards with a combination of m/z match (m/z match error < 25 ppm), MS2 spectral match (DP > 0.8) and RT match (RT match error < 60 s). These chemical structures are considered as Level 1 annotations according to MSI[7], and referred as true structures. Then, 30% of initial seed metabolites are randomly selected and used as the seed metabolites for MRN-based recursive metabolite annotation, and asked whether MetDNA could annotate the rest 70% metabolites. This experiment has been repeated 10 times. The MetDNA annotation results from the 30% of initial seed metabolites are compared to

the set of validation metabolites. For each MS1 peak in the validation set, the annotations from MetDNA are classified as correct, isomer and error. Specifically, if one of top 3 annotations from the MetDNA is the true structure, the annotation is denoted as correct. If one of top 3 annotations from MetDNA is the isomer of true structure, the annotation is denoted as isomer. If none of top 3 annotations from MetDNA is isomer of true structure, the annotation is denoted as error. In MetDNA, the annotations are ranked by their $Score_{iden}$. The random selection of 30% of initial seeds repeats 10 times, and the results are summarized in Supplementary Data 4 for *Drosophila* aging samples (dataset #1) and Supplementary Data 6 for *E. coli* (dataset #9).

Validation experiment 3

MassBank library was download from the official website of MassBank [http://www.massbank.jp]. NIST17 library was purchased from the commercial website of NIST [https://www.sisweb.com/software/ms/nist.htm]. There are 2,608 and 1,464 chemical compounds (some are metabolites) in NIST17 and MassBank library acquired on Q-TOF instruments, respectively. Then both NIST17 and MassBank were used to annotate metabolites in *Drosophila* aging datasets. For metabolite annotation, the match conditions were set as: m/z error < 25 ppm, and MS2 spectral similarity (forward DP, Methods) > 0.9. In NIST, MS2 spectra acquired using collision energy (CE) 20 V were used for match. In MassBank, MS2 spectra acquired from all CE levels were used for match. Then, the MetDNA annotation results were compared to those from NIST and MassBank libraries, and the criteria for correct, isomer and error were same with validation experiments 1 and 2.

Comparison of MetDNA and *in-silico* MS2 spectral match from CFM-ID

To benchmark and compare the performance of MetDNA and *in silico* MS2 spectra match, an experiment was designed and described below. All the metabolites in MRN (6,439 metabolites) were utilized to predict their theoretical MS2 spectral using the very popular command line tool of CFM-ID[8] [https://sourceforge.net/projects/cfm-id/] (version 2.0). The pre-trained set models (metab_se_cfm) and recommend parameters (param_output0.log) were used in both positive and negative modes, respectively. All predicted MS2 spectra were further normalized to the base peak (the highest peak intensity). For each metabolite, the 20, 30 and 50 eV MS2 spectra for positive and negative modes, respectively were generated. It means that one metabolite has 6 theoretical MS2 spectra with three collision energy values and two polarity modes.

Then we identified 200 standard metabolites in mouse liver samples using *in silico* MS2 spectra match. Manual comparison with chemical standards demonstrated that 113 and 137 metabolites were detected and

validated by MS positive and negative modes, respectively. Then the detected metabolic peaks were matched in *in silico* MS2 spectra library. The m/z error cutoff was set as 25 ppm, and the retention time (RT) cutoff was set as 30%. The dot product (DP) cutoff was set as 0.3, 0.4, 0.5, 0.6, 0.7, 0.8 and 0.9, respectively. As expected, the annotation rate decreased with the increasing cutoff of DP (Supplementary Fig. 13c). When the cutoff of DP was set as 0.5, the annotation rates were 60.2% and 62.0%, respectively (Supplementary Fig. 13c). Within this annotation rates, for the positive mode, the top 1-3 correct annotation rates for *in silico* MS2 spectral match were 57.4%, 67.6% and 70.6%, respectively. As a comparison, the correct annotation rates for MetDNA were 66.3%, 74.2% and 77.5% respectively. On the other hand, the erroneous annotation rates for *in silico* MS2 spectra match were larger than MetDNA (Supplementary Fig. 13e). The negative mode dataset had the similar results (Supplementary Fig. 13f, g and h). All these results demonstrated that MetDNA has higher correct annotation rate compared to the *in silico* MS2 spectral match using CFM-ID.

The similarities between the standard MS2 spectra (in-house library) and predicted MS2 spectra from CFM-ID were also compared and evaluated using dot product. As shown in Supplementary Fig. 13i, for the MS2 spectra in positive mode (CE=30 eV), the percentage of metabolites with DP larger than 0.5 was only 36.4%, and the mean DP and median DP were 0.40 and 0.33, respectively. The MS2 spectra in negative mode (CE=30 eV) had the similar results (Supplementary Fig. 13j). We also compared the MS2 spectra with CE=20 eV or 50 eV in both positive and negative modes (Supplementary Fig. 13k). All those results demonstrate that the accuracy for the in silico MS2 spectra still requires further improvement.

Supplementary Note 5

To investigate the influence of tandem spectral library size on the results of MetDNA, a strategy was designed and described below. For *Drosophila* aging dataset, the initial seed metabolites were selected according to the workflow of MetDNA. Then 10%, 20%, 30%, 40%, 50%, 60%, 70%, 80% and 90% of all initial seed metabolites were randomly selected from all initial seed metabolites. The different numbers of initial seed metabolites were used to mimic the different sizes of tandem spectral libraries. The result using all initial seed metabolites were considered as the control. The results obtained using different numbers of initial seed metabolites were summarized, ands compared with the control to calculated annotated metabolite number (Fig. 4a), confidence level (Supplementary Fig. 17a), and annotation redundancy (Supplementary Fig. 17b). In addition, the overlap percentages of MS1 peaks with annotations using different initial seed metabolites and the overlap percentages of MS1 peaks with the exact same annotations using different initial seed metabolites were also calculated (Fig. 4b). In both cases, the annotation result using all seed metabolites was used as a control. The similar experiment was also repeated using the aging mouse liver dataset (Supplementary Fig. 18).

The pathway enrichment results using 10%, 30%, 50%, 70% and 100% (i.e., all seed metabolites) of all initial seed metabolites were also summarized, and a Venn diagram was drawn to assess the consistence of pathway enrichment results with different size of tandem spectral library in MetDNA (Supplementary Fig. 16).

Supplementary Note 6

For positive mode of *Drosophila* aging dataset, metabolites were firstly annotated through m/z match (m/z match error < 25 ppm), measured standard RT match (RT match error < 60 s) and MS2 spectral match (dot product > 0.8). Finally, 167 peaks with 107 metabolites were annotated and recorded as the correct seed metabolites. Then the correct seed metabolites were used to construct the misannotated seed metabolites. Take type 1 misannotated seed metabolite for instance. First, the peaks with correct annotations were retrieved, and for each peak with correct annotations, its correct annotations were removed, and then the same number of metabolites from KEGG database with m/z match error > 25 ppm and RT match error > 60 s were randomly selected and assigned to this peak. Finally, there were 167 peaks with 169 misannotated annotations for type 1 misannotated seed metabolites. Similarly, type 2, 3 and 4 misannotated seed metabolites were also constructed using the different criteria provided in Supplementary Table 4. From type 1 to type 4 misannotated metabolites, the degree of similarity compared to the correct metabolite increases. Thus, they become more challenging to distinguish, and may have higher chance to propagate during the iterative process. Then MetDNA analyses were performed using the correct or misannotated seed metabolites, and we compared and validated the annotation results (Supplementary Table 4).

To investigate the reason why the propagation of misannotated metabolites is intrinsically low, for each set of corrected or misannotated seed metabolites, we first retrieved the neighbor metabolites of all the seed metabolites. Then, neighbor metabolites were match to all MS1 peaks through m/z and RT matches. Only the MS1 peaks with m/z match error < 25 ppm and RT match error < 30% were remained. MS2 spectra from these peaks were compared with surrogate MS2 spectra of neighbor metabolites (i.e., MS2 spectra from seed metabolites), and to calculate DP scores. For each neighbor metabolite matched with multiple MS1 peaks, only the maximum DP score was retained for statistics. So if one set of seed metabolites had total n neighbor metabolites, there will be n DP scores. The DP distribution were calculated for each set of correct and misannotated seed metabolites (Fig. 4f).

Supplementary Note 7

The generation of MS1 peak table for the *Drosophila* aging dataset using XCMS.

The data files (.mzXML format) of *Drosophila* aging samples can be accessed at [http://metdna.zhulab.cn/metdna/DatasetsDownload]. In each polarity, all of 20 MS files (.mzXML format) were processed using R package xcms (version 1.46.0)[4] for peak detection and alignment. The mzXML files were placed in two folders named as: W03 and W30 according to their groups. Then the data was processed using the code shown below:

```
##set the folder containing mzXML format data as the work directory
setwd("xxx")
##peak detection
f.in <- list.files(pattern = "\\.(mz[X] ML|cdf)", recursive = TRUE)
xset <- xcms::xcmsSet(f.in, method = "centWave", ppm = 15,
                      snthr = 10, peakwidth = c(5, 40), mzdiff = 0.01,
                      nSlaves = 4)
#Retention time correction
pdf('rector-obiwarp.pdf')
xsetc <-   xcms::retcor(xset, method = "obiwarp", plottype = "deviation",
                        profStep = 0.1)
dev.off()
# peak grouping
xset2 <- xcms::group(xsetc, bw = 5, mzwid = 0.015, minfrac = 0.5)
#Gap filling
xset3 <- xcms::fillPeaks(xset2)
##Peak table outputting
values <- xcms::groupval(xset3, "medret", value = "into")
values.maxo <- xcms::groupval(xset3, "medret", value = "maxo")
values.maxint <- apply(values.maxo, 1, max)
peak.table <- cbind(name = xcms::groupnames(xset3),
                    groupmat = xcms::groups(xset3),
```

```
                          maxint = values.maxint, values)
rownames(peak.table) <- NULL
write.csv(peak.table, "Peak-table.csv", row.names = FALSE)
```

As a result, a MS1 peak table named as Peak-table is generated.

Modify the Peak-table from XCMS

MetDNA requires a specific format for the MS1 peak table (.csv format). With the Peak-table generated from the XCMS, one can modify it as following:

(1) Keep columns named name, mzmed and rtmed and abundance of all samples.

(2) Rename the first three columns as name, mz and rt.

(3) Name the peak table Peak_Table_POS or Peak_Table_NEG according to its polarity.

Prepare MS2 data files

The 20 raw data files (.wiff format) in each ionization polarity were converted to mgf format using ProteoWizard [http://proteowizard.sourceforge.net/] (version3.0.6150). The parameter settings can be found in **Table 1** in Methods.

Prepare sample information file

A sample information file (.csv format) was prepared to describe the sample group information. The first column was named as sample.name, while the second one was named as group. Two group names (W03 and W30) were provided.

Process metabolomics data using MetDNA

The MS1 peak table (.csv), sample information file (.csv), and MS2 data files (.mgf) were all uploaded to our MetDNA webserver [http://metdna.zhulab.cn/] for data analysis. Positive and negative datasets were processed together. The data processing parameters for MetDNA were set as follows: Ionization polarity, Both; Liquid Chromatography, HILIC; MS Instrument, Sciex TripleTOF; Collision Energy, 30; Control Group, W03; Case Group, W30; Univariate Statistics, Student's *t*-test; Species, *Drosophila melanogaster* (fruit fly); Cutoff of *P*-value, 0.01; *P*-value Adjustment, Yes.

Supplementary Note 8

917 dysregulated peaks (FDR corrected *P*-value < 0.01) in *Drosophila* aging datasets were chosen for annotation using the MRN database (6,439 metabolites in total). For each metabolite, different adduct ions generated different m/z values (*e.g.*, $[M+H]^+$ and $[M+Na]^+$ for positive mode). The adduct ion table used in our study was provided in Supplementary Table 5 (24 and 16 adduct ions for positive and negative modes, respectively). For m/z match, m/z value for each of 917 peaks was used to match m/z values of adduct ions from all metabolites in the MRN database. It is important to note that the m/z values of adduct ions instead of accurate mass of metabolites were used. Then, the m/z error was calculated, and if the m/z error was less than the pre-set cutoff (*i.e.*, 25 ppm), this metabolite candidate with the specific adduct form was assigned to the MS1 peak as an annotation. Finally, the average annotation number per peak was calculated using the annotation results from all of 917 peaks (Supplementary Data 8).

For m/z and RT match, firstly, a total of 623 metabolites in our in-house library with measured RTs were used as the training data to construct the RT prediction model (see Methods). The RTs of these metabolites were measured using the same LC system as *Drosophila* aging datasets. With the RT prediction model, the theoretical RTs of all the metabolites in the MRN were obtained. Then, m/z value and RT for each of 917 peaks were used to match m/z values and RTs of adduct ions from all metabolites in MRN database. If the m/z and RT errors were less than the pre-set cutoff values (25 ppm for m/z and 30% for RT), this metabolite candidate with the specific adduct form was assigned to the MS1 peak as an annotation. Finally, the average annotation number per peak was calculated using the annotation results from all of 917 peaks (Supplementary Data 8).

It is worthy to note that different TOF MS instruments have different resolutions and mass accuracies. Specifically for Q-TOF MS in our study (Sciex TripleTOF and Agilent Q-TOF), the resolution and mass accuracy is m/z dependent. The low mass range (*e.g.*, 50-400 Da) has a significantly lower resolution and mass accuracy than high mass range (*e.g.*, 800-1200 Da). In addition, low ion intensity may also have a detrimental effect on mass accuracy. Therefore, it is best to use a wider mass tolerance (*i.e.*, 25 ppm in our work) than the theoretical tolerance of an instrument to cover all possible situations (*i.e.*, relative low-resolution TOF instrument, low mass ion, low-intensity, *etc.*). However, this tolerance can be adjusted by users. The similar suggested tolerance was also given in our previously published protocol[9].

Supplementary references

1. Falkner, J.A., Falkner, J.W., Yocum, A.K. & Andrews, P.C. A spectral clustering approach to MS/MS identification of post-translational modifications. *J. Proteome Res.* **7**, 4614-4622 (2008).
2. Moorthy, A.S., Wallace, W.E., Kearsley, A.J., Tchekhovskoi, D.V. & Stein, S.E. Combining Fragment-Ion and Neutral-Loss Matching during Mass Spectral Library Searching: A New General Purpose Algorithm Applicable to Illicit Drug Identification. *Anal. Chem.* **89**, 13261-13268 (2017).
3. Watrous, J. et al. Mass spectral molecular networking of living microbial colonies. *Proc. Natl. Acad. Sci. U. S. A.* **109**, E1743-1752 (2012).
4. Smith, C.A., Want, E.J., O'Maille, G., Abagyan, R. & Siuzdak, G. XCMS: processing mass spectrometry data for metabolite profiling using nonlinear peak alignment, matching, and identification. *Anal. Chem.* **78**, 779-787 (2006).
5. Tsugawa, H. et al. MS-DIAL: data-independent MS/MS deconvolution for comprehensive metabolome analysis. *Nat. Methods* **12**, 523-526 (2015).
6. Cai, Y. & Zhu, Z.J. A High-Throughput Targeted Metabolomics Workflow for the Detection of 200 Polar Metabolites in Central Carbon Metabolism. *Methods Mol. Biol.* **1859**, 263-274 (2019).
7. Sumner, L.W. et al. Proposed minimum reporting standards for chemical analysis Chemical Analysis Working Group (CAWG) Metabolomics Standards Initiative (MSI). *Metabolomics* **3**, 211-221 (2007).
8. Allen, F., Greiner, R. & Wishart, D. Competitive fragmentation modeling of ESI-MS/MS spectra for putative metabolite identification. *Metabolomics* **11**, 98-110 (2014).
9. Zhu, Z.J. et al. Liquid chromatography quadrupole time-of-flight mass spectrometry characterization of metabolites guided by the METLIN database. *Nat. Protoc.* **8**, 451-460 (2013).
