# Supplementary material for: Metabolic reaction network-based recursive metabolite annotation for untargeted metabolomics
Source: Nat Commun. 2019 Apr 3;10:1516. doi: 10.1038/s41467-019-09550-x (PMC6447530; doi:10.1038/s41467-019-09550-x)
Supplement: Supplementary file 3 — Description of Additional Supplementary Files [file 41467_2019_9550_MOESM3_ESM.docx]

**Description of Additional Supplementary Files**

**File Name:** Supplementary Data 1

**Description:** The information of 200 standards spiked in mouse liver samples.

**File Name:** Supplementary Data 2

**Description:** Validation results of MetDNA in mouse liver samples in validation experiment 1.

**File Name:** Supplementary Data 3

**Description:** Confirmation of metabolite structures using commercial standards in *Drosophila* aging dataset (dataset #1).

**File Name:** Supplementary Data 4

**Description:** Validation results of MetDNA in *Drosophila* aging datasets in validation experiment 2.

**File Name:** Supplementary Data 5

**Description:** Confirmation of metabolite structures using commercial standards in *E. coli* dataset (dataset #9).

**File Name:** Supplementary Data 6

**Description:** Validation results of MetDNA in *E. coli* dataset (dataset #9) in validation experiment 2.

**File Name:** Supplementary Data 7

**Description:** Validation results of MetDNA in *Drosophila* aging datasets in validation experiment 3.

**File Name:** Supplementary Data 8

**Description:** The annotation results of 917 dysregulated peaks in Drosophila aging datasets using the MRN database according to m/z match or m/z and RT match.

**File Name:** Supplementary Data 9

**Description:** The KEGG compounds downloaded using the R package KEGGREST.

**File Name:** Supplementary Data 10

**Description:** The RPAIR databases downloaded using the R package KEGGREST.

**File Name:** Supplementary Data 11

**Description:** Information of metabolic reaction network in MetDNA.

**File Name:** Supplementary Data 12

**Description:** Information of metabolic pathways in MetDNA.

**File Name:** Supplementary Data 13

**Description:** Information of standard MS2 spectral library in MetDNA.

**File Name:** Supplementary Data 14

**Description:** Quantitative gene data of *Drosophila* aging samples.
