# Supplementary material for: Metabolic reaction network-based recursive metabolite annotation for untargeted metabolomics
Source: Nat Commun. 2019 Apr 3;10:1516. doi: 10.1038/s41467-019-09550-x (PMC6447530; doi:10.1038/s41467-019-09550-x)
Supplement: Supplementary file 18 — Reporting Summary [file 41467_2019_9550_MOESM18_ESM.pdf]

natureresearch

Corresponding author(s): Zheng-Jiang Zhu

Last updated by author(s): Feb 20, 2019

Reporting Summary

Nature Research wishes to improve the reproducibility of the work that we publish. This form provides structure for consistency and transparency in reporting. For further information on Nature Research policies, see Authors & Referees and the Editorial Policy Checklist.

Statistics

For all statistical analyses, confirm that the following items are present in the figure legend, table legend, main text, or Methods section.

n/a	Confirmed	
☐	☒	The exact sample size (*n*) for each experimental group/condition, given as a discrete number and unit of measurement
☐	☒	A statement on whether measurements were taken from distinct samples or whether the same sample was measured repeatedly
☐	☒	The statistical test(s) used AND whether they are one- or two-sided<br>*Only common tests should be described solely by name; describe more complex techniques in the Methods section.*
☐	☒	A description of all covariates tested
☐	☒	A description of any assumptions or corrections, such as tests of normality and adjustment for multiple comparisons
☐	☒	A full description of the statistical parameters including central tendency (e.g. means) or other basic estimates (e.g. regression coefficient) AND variation (e.g. standard deviation) or associated estimates of uncertainty (e.g. confidence intervals)
☐	☒	For null hypothesis testing, the test statistic (e.g. *F*, *t*, *r*) with confidence intervals, effect sizes, degrees of freedom and *P* value noted<br>*Give P values as exact values whenever suitable.*
☒	☐	For Bayesian analysis, information on the choice of priors and Markov chain Monte Carlo settings
☒	☐	For hierarchical and complex designs, identification of the appropriate level for tests and full reporting of outcomes
☐	☒	Estimates of effect sizes (e.g. Cohen's *d*, Pearson's *r*), indicating how they were calculated

Our web collection on statistics for biologists contains articles on many of the points above.

Software and code

Policy information about availability of computer code

Data collection	No software was used for data collection.
Data analysis	A detailed description of open software, code and MetDNA used in this study has been included in the Methods. The code for XCMS processing, parameters for MS-DIAL and MetDNA processing used in this study are provided in Supplementary.

For manuscripts utilizing custom algorithms or software that are central to the research but not yet described in published literature, software must be made available to editors/reviewers. We strongly encourage code deposition in a community repository (e.g. GitHub). See the Nature Research guidelines for submitting code & software for further information.

Data

Policy information about availability of data

All manuscripts must include a data availability statement. This statement should provide the following information, where applicable:

- Accession codes, unique identifiers, or web links for publicly available datasets
- A list of figures that have associated raw data
- A description of any restrictions on data availability

The metabolomics datasets of Drosophila aging can be accessed at MetaboLights (Project ID: MTBLS612 for positive and MTBLS615 for negative modes, respectively). The metabolomics datasets of mouse liver tissues can be accessed at MetaboLights (Project ID: MTBLS601 for positive and MTBLS606 for negative modes, respectively).

Field-specific reporting

Please select the one below that is the best fit for your research. If you are not sure, read the appropriate sections before making your selection.

☒ Life sciences ☐ Behavioural & social sciences ☐ Ecological, evolutionary & environmental sciences

For a reference copy of the document with all sections, see nature.com/documents/nr-reporting-summary-flat.pdf

Life sciences study design

All studies must disclose on these points even when the disclosure is negative.

Sample size	All of the experiments described in this study were performed with 6-20 samples for each group. The sample sizes were chosen because they are standard number of in most biological studies.
Data exclusions	No samples were excluded from analysis in this study.
Replication	Each experiment presented in this study was repeated in multiple sample (between 6 and 20 for each group per experiment).
Randomization	For fruit fly (dataset #1) and mouse liver datasets (dataset #2, 3 and 4), animals were assigned randomly to young (3 days for fruit flies and 24 weeks for mouses) and old groups (30 days for fruit flies and 72 weeks for mouses). For other datasets, the randomization is not required, because the samples were grouped according to their intrinsic characteristics (gene knock-out, gene mutant, gene knock-in, colorectal cancer and esophagus cancer).
Blinding	The data presented did not require the use of blinding.

Reporting for specific materials, systems and methods

We require information from authors about some types of materials, experimental systems and methods used in many studies. Here, indicate whether each material, system or method listed is relevant to your study. If you are not sure if a list item applies to your research, read the appropriate section before selecting a response.

Materials & experimental systems

n/a	Involved in the study	
☒	☐	Antibodies
☐	☒	Eukaryotic cell lines
☒	☐	Palaeontology
☐	☒	Animals and other organisms
☒	☐	Human research participants
☒	☐	Clinical data

Methods

n/a	Involved in the study	
☒	☐	ChIP-seq
☒	☐	Flow cytometry
☒	☐	MRI-based neuroimaging

Eukaryotic cell lines

Policy information about cell lines

Cell line source(s)	MEF cell lines were from Prof. Junying Yuan's Lab (Chinese Academy of Sciences, Shanghai).
Authentication	Authentication was done using STR (Short Tandem Repeat) analysis.
Mycoplasma contamination	The cell lines were not tested for mycoplasma contamination.
Commonly misidentified lines (See ICLAC register)	No commonly misidentified cells were used.

Animals and other organisms

Policy information about studies involving animals; ARRIVE guidelines recommended for reporting animal research

Laboratory animals	Brain tissues of wild-type male fruit flies (FlyBase ID: FBst0005905) were used in this study at day 3 (3-day) and day 30 (30-day). Aging mouse liver tissues (c57BL/6J; 24-week vs. 78-week; n = 10 for each group) were collected in this study. MEF cells (Mouse Embryonic Fibroblasts ,wild-type and RIP1-/- ; n = 6 for each group) were used in this study. C. elegans (wild-type vs. daf-2 mutant, 8 days, n = 6 for each group) were used in this study. E. coli samples (wild-type vs. E. coli with α-syn expression, n = 10 for each group) were used in this study. More detailed information of animals used in this study have been included in the Methods section and Supplementary Note 3 of this manuscript.

Wild animals	The study did not involve any wild animals.
Field-collected samples	The study did not involve samples collected from the field.
Ethics oversight	No ethical approval was required for fruit flies.

Note that full information on the approval of the study protocol must also be provided in the manuscript.
